# Photocatalytic Dye Degradation from Textile Wastewater: A Review

**DOI:** 10.1021/acsomega.4c00887

**Published:** 2024-05-10

**Authors:** Sadia Khan, Tayyaba Noor, Naseem Iqbal, Lubna Yaqoob

**Affiliations:** † School of Chemical and Materials Engineering (SCME), National University of Sciences and Technology (NUST), Islamabad 44000, Pakistan; ‡ U.S.−Pakistan Center for Advanced Studies in Energy (USPCAS-E), National University of Sciences and Technology (NUST), H-12 Campus, Islamabad 44000, Pakistan; § School of Natural Sciences (SNS), National University of Sciences and Technology (NUST), Islamabad 44000, Pakistan

## Abstract

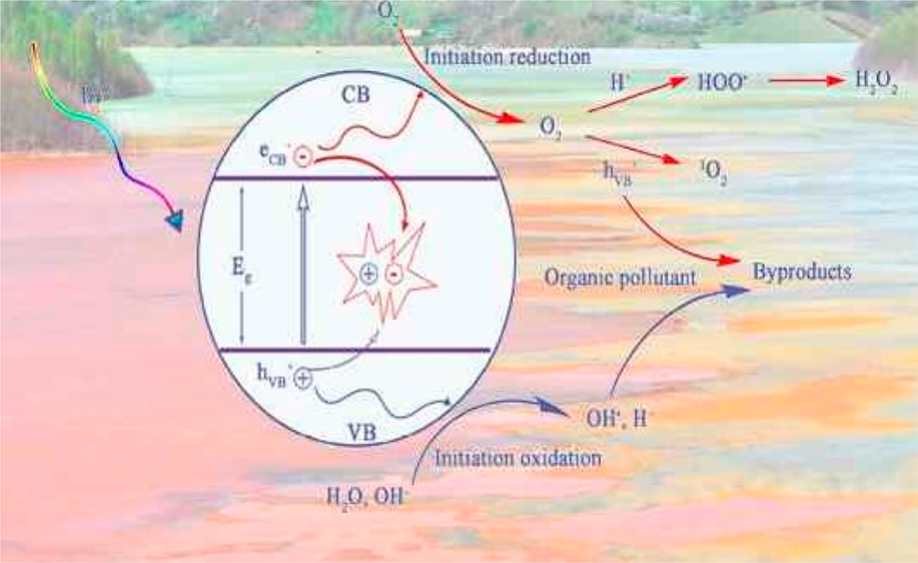

The elimination of dyes discharged from industrial wastewater into water bodies is crucial due to its detrimental effects on aquatic organisms and potential carcinogenic impact on human health. Various methods are employed for dye removal, but they often fall short in completely degrading the dyes and generating large amounts of suspended solids. Hence, there is a critical need for an efficient process that can achieve complete dye degradation with minimal waste emission. Among traditional water treatment approaches, photocatalysis stands out as a promising method for degrading diverse toxic and organic pollutants present in wastewater. In this review, the heterogeneous photocatalysis process is well explained for dye removal. This comprehensive review not only provides insightful illumination on the classification of dyes but also thoroughly explains various dye removal methods and the underlying mechanisms of photocatalysis. Furthermore, factors which effect the activity of the photocatalysis process are also explained in detail. Likewise, we categorized the heterogeneous photocatalyst in three generations and observed their activity for dye removal. This review also addresses the challenges and effectiveness of this promising field. Its primary aim is to offer a comprehensive overview of the photocatalytic degradation of pollution and to explore its potential for further future applications.

## 1 Introduction

The textile industry is one of the most prominent and mature sectors in many countries, such as Pakistan, Srilanka, India, and China, but it faces the challenge of unplanned production of wastewater effluent. During the wet fabric manufacturing process, in which different stages are involved, such as printing, dyeing, and finishing, an excessive amount of water (120–280 L) and a variety of dyes are used to accomplish the process. These synthetic pigments (dyes) are produced by coal and petroleum intermediates with an estimated annual production of 7 × 10^5^ tons. In the course of a dying process, all dyes are not firmly attached to the fabric, and almost 11–15% are discharged into the water as industrial effluent, with a rise of chemical oxygen demand (COD) and biological oxygen demand (BOD).^1,2^ Therefore, to protect consumers by preventing and minimizing environmental damage, the Ecological and Toxicological Association of the Dyestuff Manufacturing Industry (ETAD) was established in 1974 by completely collaborating with the government on issues relating to the ecological effect.^3^ According to ETDA, mostly reactive dyes were present in the industrial effluents. Quansah et al. reported that crystal violet and methylene blue are protein-based pigments which are nondegradable in nature.^4^ These reactive dyes are harmful to human and aquatic life when flushed into the water and produce a carcinogenic effect as well as disturb the reproductive system.^5^ Furthermore, wastewater also affects crops when used for irrigation systems; therefore, proper treatment of wastewater is essential before discharging it into the environment.

Conventional dye removal methods (physical and biological processes) are insufficient for removing all dyes, especially reactive dyes. These dyes have more than one functional group, forming rigid bonding with the substrate and creating hindrances for degradation. Therefore, an advanced oxidation process (AOP) is used to remove the soluble dyes which cannot be removed through traditional methods. In AOP, photocatalysis is a promising technique to remove organic pollutants by oxidation. During the photocatalysis, the source of light and catalysts are used to accelerate the process of the degradation of organic pollutants. According to the source data, photocatalysis can remove 70–80% of pigments from industrial effluent. Several researchers are working on the heterogeneous photocatalysts such as ZnO, TiO_2_, Cds, and WO_3_ for dye degradation.^6^

The mechanism of photocatalysis is divided into classes: (1) Homogeneous Photocatalysis, in which reactant and catalyst are both in the same phase, usually in the solution phase. In homogeneous photocatalysis, the catalyst, often a transition metal complex or a metal-containing compound such as ruthenium or iridium complexes, absorbs light and undergoes a photochemical transformation, leading to the generation of reactive species. These reactive species, such as radicals or excited states, then initiate chemical reactions with the organic molecules present in the solution. The advantage of homogeneous photocatalysis lies in the direct and rapid interaction between the catalyst and the reactants in the same phase.^7^ (2) Heterogeneous Photocatalysis, a process where the photocatalyst and the reactants are in different phases. Typically, the photocatalyst is a solid material, such as titanium dioxide (TiO_2_), zinc oxide (ZnO), or other metal oxides, while the reactants are in the liquid or gaseous phase. This type of photocatalysis involves the absorption of light by the solid photocatalyst, leading to the generation of electron–hole pairs and subsequent redox reactions with adsorbed species on the catalyst.^8^

Among classes of photocatalysis, heterogeneous photocatalysis is superior as compared to homogeneous photocatalysis for dye removal from wastewater due to several reasons. First, the ability to recover and reuse photocatalysts makes heterogeneous systems more cost-effective and environmentally sustainable for large-scale applications. Additionally, heterogeneous photocatalysis exhibits enhanced efficiency and selectivity due to the direct interaction between the solid catalyst and dye molecules, leading to higher yields of degradation products and reduced side reactions. The stability and durability of solid photocatalysts further contribute to their superiority, as they can withstand harsh reaction conditions without significant degradation. This results in longer catalyst lifetimes and improved process sustainability. Moreover, the reduced environmental impact of heterogeneous photocatalysis with minimal contamination of the effluent by catalyst residues ensures compliance with regulatory standards. Lastly, the scalability and industrial applicability of heterogeneous systems make them suitable for commercial dye removal applications in wastewater treatment facilities.^9−12^

## 2 Classification of Synthetic Dyes

Synthetic dyes are artificially produced colorants used in various industries, including textiles, cosmetics, and food. They are created through chemical processes and are often more vibrant and stable than natural dyes. Synthetic dyes are classified into ionic and nonionic compounds based on their chemical properties and application method.^13^

**Figure 1:**
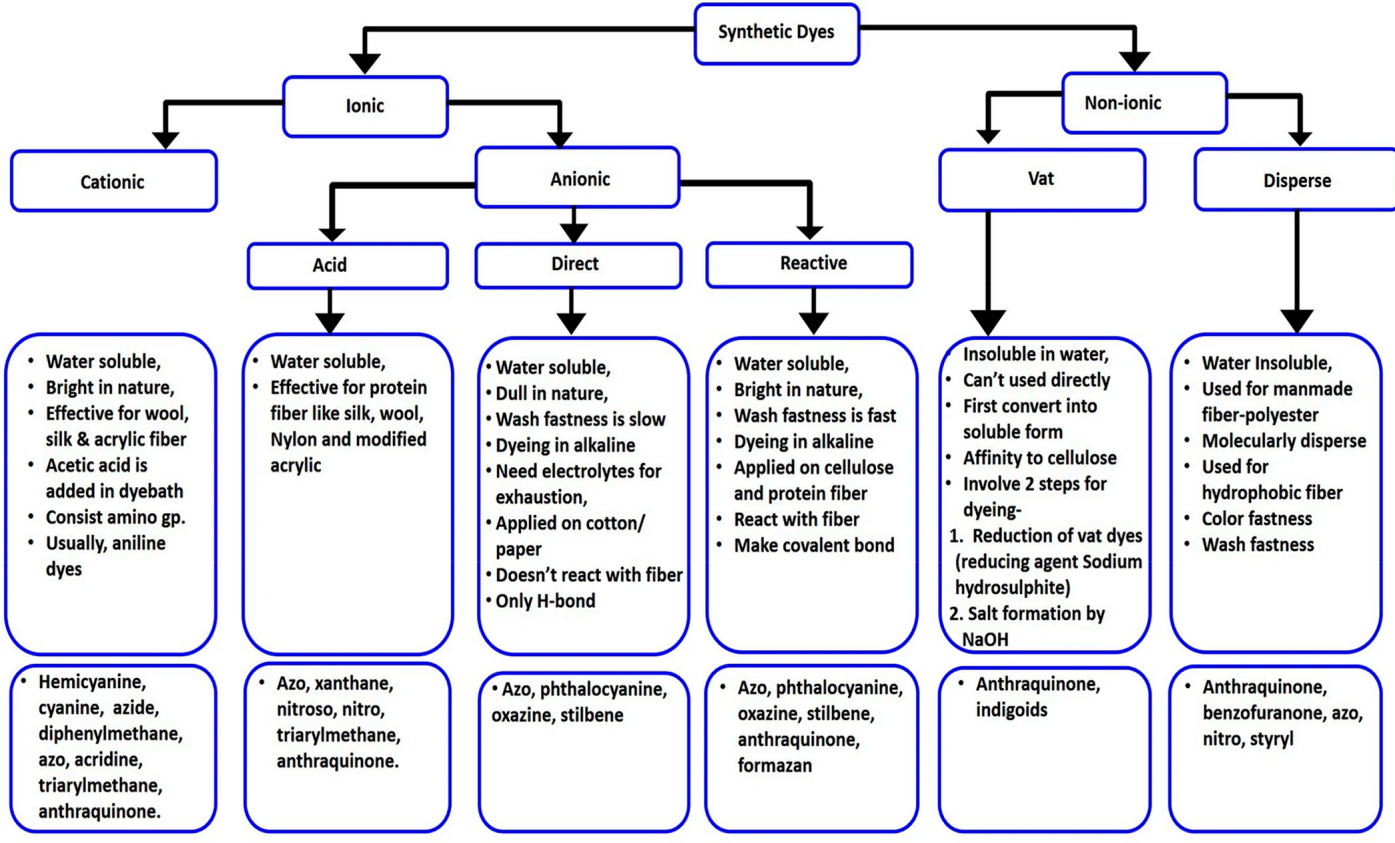
Classification of synthetic dyes. Reproduced with permission from ref (14). Copyright 2023, Springer Nature.

In between the two categories are ionic and nonionic dyes (as shown in Figure 1). Ionic compounds contain cationic or anionic charge ions, and cationic dyes carry a positive charge and are commonly used to color materials with negatively charged surfaces, such as acrylic fibers and certain types of paper. They establish electrostatic attractions with negatively charged groups on the substrate, resulting in robust bonding. These dyes interact with the substrate through ionic interactions, forming strong bonds with materials that possess opposite charges. Anionic dyes carry a negative charge and are often employed to color materials with positively charged surfaces such as natural fibers like cotton and wool. They form bonds with positively charged groups on the substrate through electrostatic interactions.^14,15^ In contrast, nonionic dyes do not possess a net charge on their molecules. Instead, they rely on weaker forces such as van der Waals forces, hydrogen bonding, and hydrophobic interactions to adhere to the substrate. Nonionic dyes are frequently utilized for dyeing materials with neutral surfaces or surfaces that do not readily interact with charged molecules. They offer versatility in terms of substrate compatibility and can be used across a wide range of materials. Examples of nonionic dyes include dispersed dyes, which are commonly used to color synthetic fibers like polyester and nylon, and vat dyes, which are employed for dyeing natural fibers such as cotton and linen. Ionic dyes are commonly used in dyeing processes and can have significant impacts on water quality when discharged as wastewater.^16,17^ The discharge of dye-containing wastewater from industrial processes poses significant threats to aquatic ecosystems. Synthetic dyes can lead to water contamination, alter their physical, chemical, and biological properties, and render them unsuitable for various uses, including drinking water sources and aquatic habitats. Additionally, highly pigmented dyes can reduce light penetration into water bodies, disrupting photosynthesis and oxygen levels and thereby affecting overall ecosystem health. Certain classes of synthetic dyes, such as azo dyes (−N=N−), exhibit toxicity to aquatic organisms, leading to adverse effects on fish, invertebrates, and other life forms. Moreover, synthetic dyes can alter water chemistry parameters, disrupt nutrient cycling processes, and persist in the environment for extended periods, posing ongoing risks to aquatic ecosystems. Effective wastewater treatment processes, regulatory measures, public awareness campaigns, and eco-friendly dyeing techniques are essential for mitigating the environmental impact of synthetic dyes on water resources.^18−21^

## 3 Fundamental Techniques for Dye Degradation

Multiple techniques, i.e., biological, physical, and advanced oxidation methods, are utilized for the degradation of dyes from the textile wastewater; their strength and limitation concerning dye degradation are mentioned in Table 1.

**Table 1:** Strengths and Limitations of Different Techniques for the Dye Removal

Techniques	Strength	Limitation	Ref
**Physical Method**	Good for removal of insoluble dyes	Not applicable to all dyes	(22−26)
Adsorption	Low operational cost	The high cost of some activated carbon	
Membrane Filtration	Effective at lab scale	Inadequate quality for reusing the permeate	
Nanofiltration	No adsorbent loss (regeneration)	Disposal of suspended effluent is difficult	
Microfiltration			
Ion Exchange			
Coagulation			
Reverse Osmosis			
**Biological Method**	Enzymes are used for the degradation of dyes	Low removal rate	(27, 28)
Micro-organism	Eco-friendly process	Cannot degrade all types of dyes	
Plant	Economically attractive method, no additional operation cost is required		
Enzymes	Less sludge is produced as compared to other methods	Large space is required	
	Effective for azo dyes		
**Chemical Method/Advanced Oxidation Process**	Quick and efficient process	Sludge is produced during the process	(29−32)
Ozonation	Good elimination of pigments	High cost of electricity required	
Fenton	Efficient for soluble and nonsoluble dyes	Producing aromatic chemicals, which can be harmful to human health and the environment	
UV Hydrogen Peroxide	Electrons are used for the degradation of pigments		
Photocatalysis			

The aforementioned information makes it clear that biological techniques are quite famous because of their economic nature, but this technique is kinetically slow and cannot remove all of the dyes from wastewater. Similarly, physical techniques like adsorption, flocculation, precipitation, reverse osmosis, and coagulation are also not very effective to remove all of the dyes from the wastewater.^33^ However, the advanced oxidation process (AOP) holds promise which can degrade the ionic and nonionic nature of dyes from wastewater. AOP is characterized by its nonselective nature, making it effective in degrading a wide range of organic pollutants, through the generation of highly reactive hydroxyl radicals (OH^•–^). These hydroxyl radicals react rapidly with organic compounds, breaking down complex dye molecules into smaller, less harmful byproducts. AOP offers advantages such as (1) high efficiency, (2) versatility, and (3) applicability to various wastewater matrices, making it a promising approach for the treatment of dye-containing wastewater. The AOP constitutes a diverse array of methodologies designed for the degradation of organic pollutants, particularly synthetic dyes, within wastewater treatment. AOP incorporates both homogeneous and heterogeneous chemical reactions, where the catalyst and reactants may be in the same or different phases, respectively. Fenton processes, a part of AOP, leverage iron salts and hydrogen peroxide to generate highly reactive hydroxyl radicals (OH^•–^), which effectively break down organic compounds. Additionally, electrochemical oxidations involve the application of an electrical current to drive oxidation reactions and produce reactive species such as hydroxyl radicals. Photolysis, using both gamma and ultraviolet (UV) irradiation, plays a significant role in AOP.^34−37^

Photocatalysis has emerged as a particularly superior technique for the degradation of organic pollutants, including synthetic dyes, in wastewater treatment. Photocatalysis harnesses the power of catalysts, such as titanium dioxide (TiO_2_), under UV irradiation to generate highly reactive hydroxyl radicals (OH^•–^). This process exhibits several advantages over conventional wastewater treatment techniques. First, photocatalysis offers exceptional efficiency in degrading organic pollutants due to the rapid generation of hydroxyl radicals upon exposure of light. Second, it operates under relatively mild conditions of low temperature and pressure, minimizing energy consumption and operational costs. Third, photocatalysis produces no harmful side products, ensuring environmentally friendly treatment of wastewater. Additionally, photocatalytic processes are readily scalable and adaptable to various wastewater matrices, making them applicable in diverse industrial and environmental settings. These inherent advantages position photocatalysis as a leading technology for the removal of synthetic dyes and other organic pollutants from wastewater, offering a sustainable and efficient solution to water contamination issues.^38−40^

## 4 General Mechanism of Photocatalysis for Dye Degradation

Photocatalysis is a process where a photocatalyst (TiO_2_) absorbs photons (typically ultraviolet light) to generate electron–hole pairs, which then participate in redox reactions with adsorbed species on the catalyst surface, leading to the degradation of synthetic dyes in wastewater. The general mechanism of photocatalysis involves three steps:^41−43^

(1) Generation of electron–hole pairs: When the photocatalyst absorbs photons with energy greater than its bandgap, electrons are excited from the valence band to the conduction band, leaving behind positively charged holes in the valence band.
(2) Redox reactions: The electrons in the conduction band and the holes in the valence band are highly reactive and can participate in redox reactions with adsorbed species on the catalyst surface, such as water and oxygen, or organic pollutants.
(3) Degradation of pollutants: The reactive species, such as hydroxyl radicals (OH^•–^) formed from water molecules, can react with organic pollutants adsorbed on the catalyst surface, leading to their degradation into smaller, less harmful byproducts.

The surface of the catalyst also plays a major role in the removal of dye. As the surface of the catalyst is large, the maximum amount of dye degraded during the photolysis. The decomposition of dyes on the surface of the catalyst can be done by two pathways: one is the direct photocatalytic mechanism and the second is the indirect photocatalytic mechanism.

### 4.1 Direct Photocatalytic Mechanism

Two methods have been suggested to explain the direct mechanism, the Langmuir–Hinshelwood method and the Eley–Rideal method.

**Figure 2:**
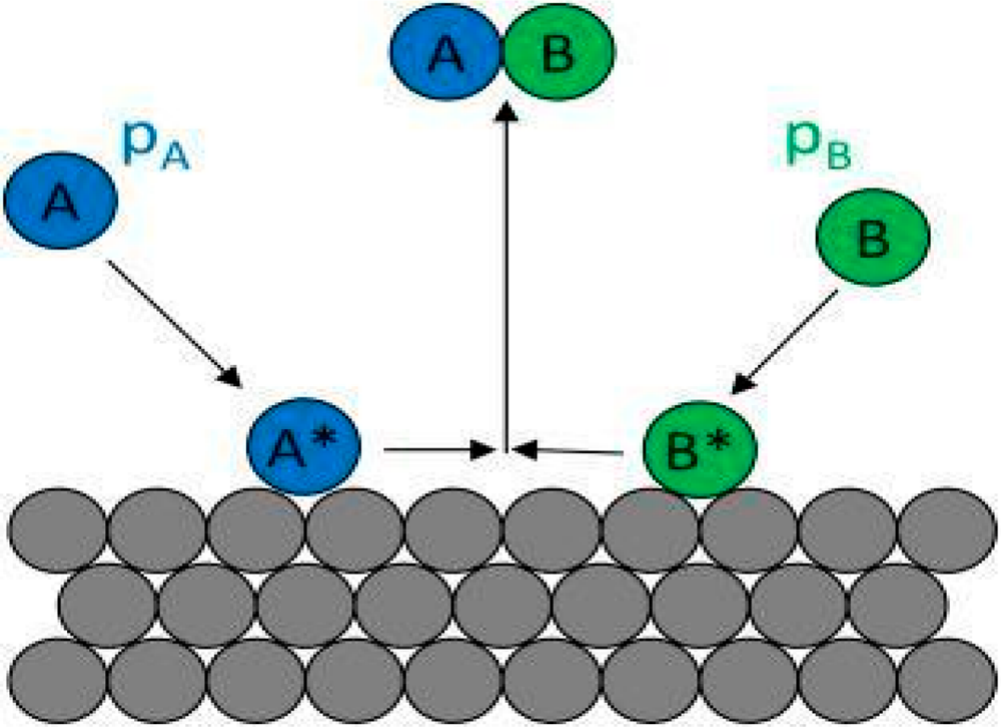
Representation of the adsorption phenomena by the Langmuir–Hinshelwood mechanism. Replicated with permission from ref (44). Copyright 2018, Elsevier.

In 1921 Langmuir was the first one who described the adsorption theory, and then in 1926 Hinshelwood refined this theory, and the adsorption theory also known as the Langmuir–Hinshelwood theory^44^ is presented in Figure 2. It explains that both the electron and hole are produced by photoexcitation of the catalyst, and they react with the stuck dye molecule on the catalyst surface and form active radical species followed by dye decomposition after the combination with the electron and catalyst is regenerated.^44,45^

The Langmuir–Hinshelwood equation to find the rate of degradation is:^46^
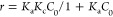
where *K*_a_ is the adsorption constant; *K*_c_ is the specific rate constant; and *C*_0_ is the initial concentration.

**Figure 3:**
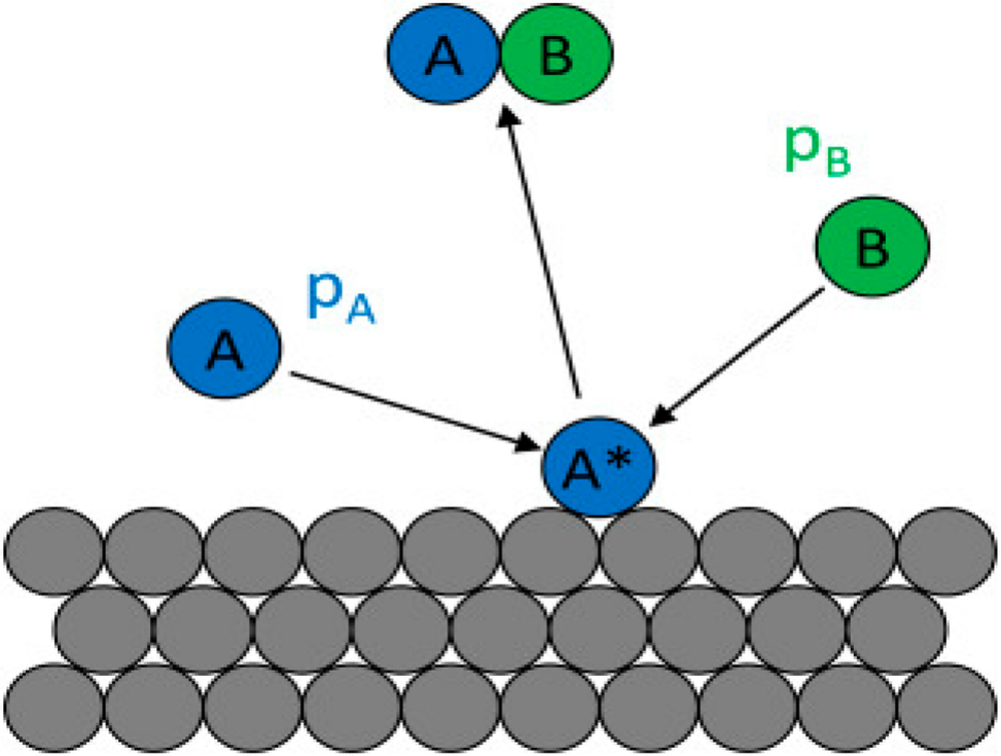
Representation of the adsorption phenomena by the Eley–Rideal method mechanism. Replicated with permission from ref (44). Copyright 2018, Elsevier.

In 1938, Eley–Rideal described the second method, as shown in Figure 3. In this method, first the hole was trapped on the surface of the defect, and then photoexcited species react with dye which becomes an intermediate species. The intermediate compound further spoils the products or recombines with electrons.^44^

The reaction scheme for dye removal is given below^45,47,48^
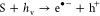

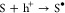

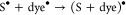

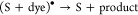
In this reaction, *h*_v_ is the intensity of light; “S” is the surface of the catalyst; and e^•–^ and h^•^ are the electron and hole respectively, where the dot ^•^ represents the radical.^49,50^

### 4.2 Indirect Photocatalytic Mechanism

**Figure 4:**
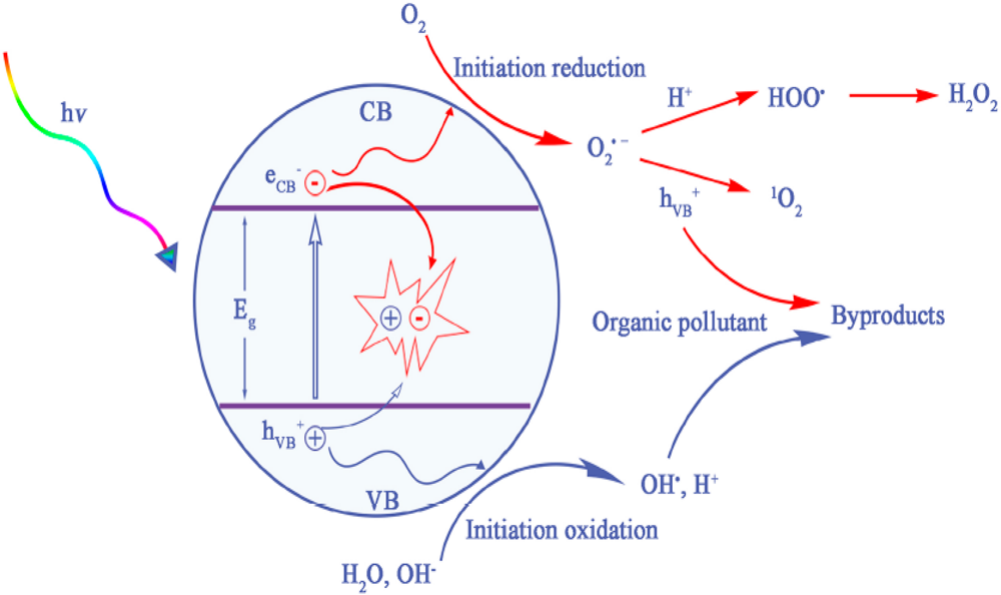
Mechanism of dye degradation occurring on an ideal photocatalyst imbued with dye reduction and oxidation reaction. Reproduced with permission from ref (53). Copyright 2020, Elsevier.

In the second method, the electron and hole are both photogenerated on the surface of the catalyst as shown in Figure 4. When the beam of light (UV or solar) strikes on the surface of the catalyst, it absorbs the photon energy greater than its band gap which generates electron (e^•–^) and hole (h^+^) bands, as represented below^51^
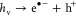
This mechanism is comprised of the following steps:

(1) Photoexcitation: The semiconductor consists of the valence band (VB) and conductance band (CB). The difference is called the energy gap (*E*_g_). When the beam of light (photon) strikes with a semiconductor, the photoelectron is transferred from the valence band to the conduction band as a result of absorption of ultraviolet radiations. Due to this photoexcitation, a hole (h^+^) is generated on the valence band and a charge carrier (e^•–^) on the conduction band.
(2) Dissociation of water: On the valence band, OH^•–^ is generated as a result of the reaction of photogenerated holes with water molecules. The irradiated semiconductor surface acts as a powerful oxidizing agent, and organic pollutants present on the dyed surface chemically react with OH^•–^ radicals and formulate a chain of radicals by consuming oxygen and after decomposition of organic matter into carbon dioxide and water.
(3) Oxygen ion sorption: During the formation of hydroxyl radical molecules by the reaction of surface bound water and photogenerated electrons, molecular oxygen fills up the conduction band and produces an anionic superoxide radical. This superoxide not only takes part in the oxidation process but also prevents the recombination of the electron–hole.
(4) Protonation process of the superoxide: The reduction process involves the easy reduction of oxygen and the production of hydrogen. The electrons present on the conduction band react with dissolved O_2_^•–^ and form superoxide anions, which then attached to the intermediate product produced from oxidative reaction and resulted in peroxide or converted them into an ultimate product of hydrogen peroxide and H_2_O.^52^

The detailed mechanism of photolysis in the form of an equation is represented as^54−59^
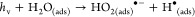

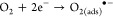

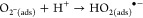

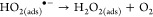

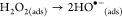


Finally, the dye reacts with an OH radical and for intermediate (dye degradation) and end products.
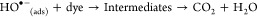


The mathematical form to find out the % age degradation of dyes is^60^
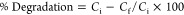
where *C*_i_ is the initial concentration of dye before degradation, and *C*_f_ is the final concentration of dye after degradation.

## 5 Parameters Affecting the Photocatalytic Dye Degradation

There are different parameters affecting the process of degradation of dyes such as pH, temperature, intensity of light, dose of photocatalyst, initial concentration of dye, shape of the catalysts, etc.

### 5.1 Effect of pH

In the process of the degradation of dye by heterogeneous photocatalysis, pH is a critical operating parameter that affects (a) the charge on the surface of a photocatalyst, (b) the charge on a dye molecule, (c) photocatalyst aggregate size, and (d) the positions of the valence band and conductance band. The pH of polluted water contaminated by dye regulates the electrostatic interaction present between the substrate, catalyst surfaces, and radicals generated during the degradation process.

Chen et al. in 2007 exposed that the adsorption of malachite green (MG) dye on the photocatalyst surface was reduced when photogenerated charge carriers recombine and accelerate the charge transfer process at interfaces.^63^ The MG is a cationic organic dye with low pH, and more H^+^ was available for adsorption and in turn masked the surface of the catalyst, preventing the photoexcitation of the semiconductor particle and reducing the generation of free radicals. So, the strong force of repulsion between the positive charge semiconductor metal oxide surface and cationic MG molecules makes it difficult for MG molecules to get adsorbed on the surface of semiconductor oxide under acidic conditions. Therefore, this repulsion decelerates the degradation efficacy of the photocatalyst at low pH. However, under basic conditions, strong adsorption of MG molecules occurs at high pH, but excessive adsorption blocks the light harvesting on the photocatalyst surface and hinders the process of photoexcitation. Gaya et al. studied the effect of change in pH on the photocatalytic oxidation proficiency of TiO_2_. They witnessed the strong oxidation performance of TiO_2_ at pH less than 2. However, the reaction rate tends to decrease when pH < 2.^61−63^

### 5.2 Effect of Temperature

As the photocatalytic process is light dependent, the lower effect of temperature is observed in the range of 20–80 °C. At temperatures below 0 °C or temperatures above 80 °C, a declining trend is observed in photocatalytic activity. Thus, photocatalytic activity does not need particular temperature control. A slight benefit effect is witnessed at a high temperature. It might be due to rapid diffusion of OH^–^ ions from the surface of the catalyst toward the pollutant. However, the dissolved oxygen concentration of the dissolved oxygen ratio in solution tends to decrease at high temperature, and this low oxygen concentration after a certain point allows electron–hole recombination on the TiO_2_ surface. Dominant electron–hole recombination takes place, except the electron acceptor like oxygen is available to absorb the photoexcited electron. Hassan et al. verified the role of temperature (25–40 °C) on the speed of anthracene degradation by using green NPs of ZnO. The degradation efficiency revealed that an incline in temperature leads to a small increase in the rate of reaction. This might be due to the amplified collision frequency of the targeted molecules. However, at higher temperatures, desorption of the contaminant from the catalyst surface can result in a low reaction rate.^64,65^

### 5.3 Efficacy under Visible Light Irradiation

Photocatalytic degradation of the dye relies on the intensity of energy delivered by the light quanta. Absorption of a photon of energy equal to/greater than the band gap generates the electron–hole pairs in the valence and conduction band. The dye removal efficiency significantly increases with the increase of irradiation power from 16 to 32 W and then slightly increases with subsequent incline from 32 to 48 W followed by the dye removal process to be independent of the light radiation intensity. Some experiments also indicate that the speed of the dye removal process becomes almost double with an increase of light intensity from 10 to 70 mW/cm^2^. In another study, a negative relation of light intensity with the degradation process was observed on the Congo Red dye in the range of 50–90 J/cm^2^. Initially the dye removal rate was high until a light intensity of up to 80 J/cm^2^, but at 90 J/cm^2^, the degradation rate tends to drop due to thermal effects linked with an increase of temperature of the dye-tainted solution.^66−68^

In 2010, Yoon et al. elaborated on the potential of light for eco-friendly chemical reactions due to its nontoxic nature, absence of waste generation, and renewable sourcing. However, they noted that traditional organic photochemical processes rely on high-energy ultraviolet radiation, limiting their practicality and environmental benefits on a large scale. The researchers explored the use of metal polypyridyl photocatalysts in organic transformations to overcome this limitation. Catalysts like Ru(bpy)_3_^2+^ were highlighted for their ability to harness visible light from sources such as fluorescent bulbs or sunlight to enhance practicality and eco-friendliness for industrial applications.^69^

In 2023, Mugumo et al. degraded Rhodamine B (RhB) dye in the presence of visible light irradiation and heterojunction nanocomposite CuS/ZnS. This results in 97% of 5 ppm RhB dye being removed after 270 min of visible light irradiation.^70^

In 2023, Alwared et al. conducted a study of the solar-induced photocatalytic degradation of reactive red and reactive turquoise dyes using TiO_2_ immobilized in xanthan gum. The research found that the catalyst exhibited a high degradation efficiency, achieving 92.5% degradation of reactive red dye and 90.8% degradation of reactive turquoise dye within 120 min under solar light.^71^

In 2024, Karuppaiya et al. conducted a study focusing on the removal of cationic (Rhodamine B) and anionic (Eosin yellow) dyes utilizing nanocomposite SnO_2_–Zr–F under solar light exposure. The research observed a significant degradation of both dyes, with a maximum degradation efficiency of 92% for cationic dye (Rhodamine B) and 98% for anionic dye (Eosin yellow) achieved over a 150 min period under sunlight.^72^

### 5.4 Effect of Photocatalyst Loaded Amount

The concentration of the photocatalyst in a wastewater has a noteworthy effect on the reaction rate as well as dye degradation capability. Increasing the loaded amount of photocatalyst generates additional electron–hole pairs and leads to a subsequently higher and faster dye degradation process. However, at higher concentrations, high turbidity of wastewater increases the light scattering tendency and decreases its penetration power in the reaction mixture. Hosseini and colleagues in 2018 reported the increased photocatalytic activity with increase of concentration, but the photocatalytic activity and decolorization efficiency started to decrease after a further increase in the amount of photocatalyst. In recent times, the negative effect of composite concentration on the degradation process was reported. The study of degradation of *para*-nitrophenol was scrutinized against different concentrations of composite dosage (0.05, 0.15, 0.25, 0.35, and 0.45 g/L), and the best removal tendency (97%) was attained at the optimized composite concentration of 0.25 g/L. The decrease in removal activity at higher concentration was due to lessened penetration and amplified light scattering. In 2015, Tahir et al. reported that with an increase of Ag NP concentration from 2–8 mg/mL the MB decomposition raised from 40 to 96%. At lower dosage, the particles are normally very small and extremely dispersed, which delivers a huge surface area with more active sites and results in increased dye degradation efficiency. A further upsurge in the catalyst amount, from 8–12 mg/mL, does not further support the degradation process due to agglomeration of large size Ag NP catalysts and consequent low surface area and reduced number of active sites.^73−75^

### 5.5 Effect of Dye Concentration

The adsorption of pollutants (dyes) on the surface of a photocatalyst depends on the binding affinity and electrostatic interactions between the catalyst and dye molecule. Adsorption of a moderate amount of dye molecules on the surface of the photocatalyst is beneficial for the process of degradation based on the synergies between photocatalysis and the adsorption process. Composite materials possessing upright adsorption properties exploit a synergistic effect for concurrent adsorption and dye degradation. However, a high concentration of adsorbed dye molecules on the surface of the catalyst overpowers the quantity of photons approaching the surface-active sites. Moreover, dye molecules can also work as sensitizers which absorb the electrons and then scatter them in an unwanted direction. The synergistic effect between these two mechanisms for methylene blue degradation was reported by Anwer and colleagues. A composite of graphene oxide with TiO_2_ caused 50% augmentation in the photodegradation of dye compared to TiO_2_ alone. The concentration of dye after the optimized amount seems to be disadvantageous for reaction.^74,76^

### 5.6 Effect of Electron Acceptors

One practical issue with the utilization of the TiO_2_ photocatalyst is the loss of energy during electron–hole recombination that results in squat degradation efficiency. Henceforth, the inhibition of electron–hole recombination was found to be very important. Molecular oxygen is normally employed as an operative e^–^ acceptor in photocatalytic applications. In heterogeneous conditions, molecular oxygen from air was selected as an electron acceptor to restrict the electron–hole recombination. Another approach to mitigate electron–hole recombination in photocatalysis is by incorporating hydrogen peroxide (H_2_O_2_) as an electron acceptor. H_2_O_2_ serves as an electron scavenger, effectively trapping electrons from the conduction band of the photocatalyst, thereby preventing their recombination with holes and enhancing the efficiency of photodegradation. This process has several beneficial effects. First, by accommodating the electrons from the conduction band, H_2_O_2_ effectively reduces the likelihood of recombination, allowing more electrons to participate in the degradation reactions. Second, H_2_O_2_ increases the concentration of hydroxyl radicals (OH^•–^) through reactions with holes or other intermediates. Hydroxyl radicals are highly reactive species and play a crucial role in the degradation of organic pollutants. In addition to H_2_O_2_, other suitable options for electron scavenging and recombination suppression include persulfate ions (S_2_O_8_^–2^) and bromate ions (BrO^–^). These species act similarly to H_2_O_2_ by capturing electrons and preventing their recombination with holes, thereby enhancing the overall photodegradation efficacy.^63,77,78^

### 5.7 Effect of Intermediate Species

The degradation of dyes through photocatalysis involves the generation of reactive oxygen species (ROS) and other intermediates, each playing a vital role in the overall process. Reactive oxygen species like hydroxyl radicals (OH^•–^), superoxide radicals (O_2_^•–^), and hydrogen peroxide (H_2_O_2_) are instrumental in breaking down dye molecules into less toxic or nontoxic compounds. However, the presence of inorganic anions, such as carbonates (CO_3_^2–^), chlorides (Cl^–^), nitrates (NO^3–^), and sulfates (SO_4_^2–^), can impact the photocatalytic degradation process by scavenging hydroxyl radicals (OH^•–^) and other ROS, thereby reducing their concentration and efficiency. Conversely, the addition of oxidizing agents such as hydrogen peroxide (H_2_O_2_), ammonium persulfate ((NH_4_)_2_S_2_O_8_), and potassium bromate (KBrO_3_) can enhance the photocatalytic degradation of dyes by providing additional oxidizing power. It is crucial, however, to carefully control the concentration of these agents to avoid inhibiting the photocatalytic process. In summary, the influence of intermediate species on photocatalytic dye degradation is intricate and contingent on specific conditions and reactants. Proper management of these factors is essential to optimize the efficiency of dye degradation and minimize the formation of undesirable byproducts. While the ideal outcome of a photocatalytic reaction is the production of water and carbon dioxide, the breakdown of organic dyes can yield intermediate molecules that may have more detrimental effects than the original dye compound. Monitoring and understanding these transitional products are crucial, as they can pose potential risks.

Under ideal situations, the photocatalytic reaction irradiated by UV light yields water and carbon dioxide as the final products. However, the organic dyes due to their large structures can be broken down into smaller molecules during the process of photocatalytic degradation, and these intermediate molecules can have a more detrimental effect than the original dye compound. Generally, the reaction intermediates are not monitored, and no work is done to explore the transitional products formed during dye degradation. Chen et al. studied the degradation of *N*,*N*,*N*,*N*-tetraethylsulforhodamine-B dye and recognized the reaction intermediates possessing a dissimilar number of *N*-ethyl groups. These reaction intermediates were radically identical with parent dye molecules. However, it would be erroneous to assume that the same situation will happen in all reactions. These transitional products can be more dangerous than the parental dye. For example, one of the side products of phenol degradation is catechol, and convulsions and hypertension developed in animals from catechol are greater than from phenol.^79,80^

### 5.8 Effect of Inorganic Ions on Photodegradation

The efficiency of the photodegradation processes is influenced by the type and concentration of inorganic ions in the solution. Inorganic ions have the potential to either enhance or inhibit the photocatalytic activity of TiO_2_, depending on their specific characteristics and concentrations. For instance, chloride (Cl^•–^) and sulfate (SO_4_^•2–^) ions have been observed to enhance the degradation efficiency of TiO_2_. Conversely, ions, such as carbonate (CO_3_^•2–^) and phosphate (PO_4_^•3–^), can impede the photocatalytic activity of TiO_2_. Furthermore, the presence of inorganic ions can also impact the adsorption of dyes onto the TiO_2_ surface, thus influencing the overall degradation process.^81^ Hu et al. performed the experiment to degrade the cationic blue (CBX) and red MX-5B which are the subclass of azo dyes using the inorganic ions SO_4_^•2–^, H_2_PO_4_^•–^, ClO_4_^•–^, and F^•–^. They observed that at pH 2.4 the decolorization rates of MX-5B and CBX are enhanced on the surface of TiO_2_. At pH 10.8, most of the selected anions inhibited the photocatalytic oxidation to decolorize and degrade CBX and MX-5B. These results demonstrated that inorganic anions affect the photodegradation of dyes by their adsorption onto the surface of TiO_2_ and trapping positive hole (h^+^) and OH. Inorganic cationic ions, such as Cu^•2+^ and Ni^•2+^, had strong inhibition on the decolorization of MX-5B at pH 10.8. In strong basic conditions (pH 10.8), the main anions in solution were HPO_4_^•2–^ and PO_4_^•3–^. These anions reacted with OH to form HPO_4_^•–^ and PO_4_^•2–^, which are somewhat less reactive. The presence of these anions had a stronger inhibition effect on the photodegradation of dyes at high pH conditions.^82^

### 5.9 Impact of Photocatalyst Morphology on Degradation

The morphology of photocatalysts significantly influences the degradation of dyes during the photocatalytic processes. Various morphological characteristics, such as surface area, porosity, crystallinity, and particle size, can impact the efficiency of dye degradation. Catalysts with high surface area-to-volume ratios generally exhibit enhanced photocatalytic activity due to the increased availability of active sites for adsorption and reaction of dye molecules. Additionally, well-defined crystalline structures with specific facets can facilitate efficient charge separation and migration, leading to improved degradation kinetics.^83,84^

In 2023, Cigdem et al. conducted a study where they designed different morphologies of zinc oxide (ZnO) nanostructures, including nanoflower (NF), nanosponge (NS), and nanourchin (NU), to investigate their photocatalytic capabilities for dye degradation. Among these morphologies, ZnO NSs exhibited notably superior performance in photocatalytic dye degradation compared to that of the others. The photocatalytic activity of ZnO nanocatalysts was influenced by factors such as defect structure, pore diameter, and crystallinity. Their research study emphasized the importance of developing ZnO catalysts with fewer core defects, increased oxygen vacancies, near band emission, larger crystallite size, and larger pore diameter to achieve optimal photocatalytic activity in dye degradation.^85^

In 2023, Das et al. conducted a study on Ga_2_O_3_ photocatalysts for dye (RhB) degradation. They designed different morphologies of Ga_2_O_3_ photocatalysts such as spherical nanoparticles and fractured nanobricks using various Gallia precursors. It was observed that the nanobricks originating from nitrate salt showed superior efficiency in degrading rhodamine B (RhB) compared to the nanospheres produced from the chloride salt. The nanobricks exhibited a degradation rate constant of 0.0394 min^–1^, while the nanospheres had a lower degradation rate constant of 0.0057 min^–1^. This disparity in degradation performance was attributed to differences in electronic band positions and morphological features of the photocatalysts. The nanobricks possessed higher specific surface area, porosity, and aspect ratio, facilitating faster degradation of RhB.^86^

### 5.10 Photocatalyst’s Resistance to Photocorrosion

The stability and durability of a photocatalyst material are crucial factors for ensuring its long-term performance and cost efficiency, particularly in applications such as water and wastewater remediation. Photocatalyst corrosion resistance denotes the ability of a photocatalyst material to withstand degradation or deterioration upon exposure to corrosive environments or during photocatalytic reactions. Corrosion in photocatalysts can arise from chemical and electrochemical reactions induced by photoactivation, resulting in the dissolution of the material. Photocorrosion is a prevalent concern for semiconducting materials like zinc oxide (ZnO), which can limit their practical utility in photocatalysis.^87^

In 2023, Warren et al. conducted a study where they doped zinc oxide (ZnO) with 1% and 2% Co, Ni, or Cu salts, resulting in a notable enhancement in stability with a significant reduction in zinc ion leaching following exposure to light. However, this modification also caused a decline in the material’s photocatalytic activity. They explained that the doping process caused a decrease in the band gap of the material and increased its resistance to photocorrosion. Nonetheless, the decrease in photocatalytic activity was attributed to alterations in the material’s energy level positions. Further research is warranted to maintain the photocatalytic efficiency of ZnO while simultaneously enhancing its stability in water.^88^

### 5.11 Strategies for Photocatalyst Regeneration

The primary aim of photocatalyst regeneration is to restore the catalytic functionality of the photocatalyst after it has been deactivated or saturated with contaminants. This regenerative process facilitates the reuse of the photocatalyst, reducing the need for frequent replacement and minimizing the generation of waste. The restoration of the photocatalyst enhances the efficiency and effectiveness of photocatalytic processes, ensuring continuous and sustainable operation. In the specific context of water purification, where the photocatalyst interacts with pollutants, regeneration becomes particularly vital to revive the catalyst to an active state for the ongoing degradation of pollutants.^89,90^

In 2014, Miranda et al. investigated the photocatalytic degradation of emerging contaminants using TiO_2_ photocatalysts immobilized on glass spheres. Their study involved evaluating the performance of these photocatalysts over four cycles and exploring methods for recovering the photoactivity. The results revealed the efficient removal of fluorine-containing contaminants, although compounds with amine or amide groups exhibited lower degradation rates. Regeneration techniques utilizing H_2_O_2_/UV or calcination were found to be effective in restoring photocatalytic activity. The study also underscored the preferential adsorption of certain compounds on the active sites of the photocatalyst.^91^

In 2022, Kim et al. explored the use of TiO_2_-coated zeolite (TiO_2_/zeolite) as a photoregenerative adsorbent for volatile organic compound (VOC) filters, aiming to prolong their service life. Through their experiments, they found that the TiO_2_/zeolite filter demonstrated a photoregeneration efficiency exceeding 90% during the initial two regeneration cycles under ultraviolet (UV) illumination. The presence of TiO_2_ on zeolite significantly contributed to enhancing the regeneration efficiency of VOC filters based on zeolite. Compared to filters solely composed of bare zeolite, the TiO_2_/zeolite filters exhibited notably improved regeneration efficiency, suggesting their potential suitability as adsorbents for UV-regenerative air filtration systems capable of multiple uses. Additionally, the TiO_2_/zeolite filter maintained a photoregeneration efficiency of over 60% for up to five cycles, further supporting its candidacy as a promising adsorbent for photoregenerative VOC filters.^92^

In their 2022 study, Yong et al.^93^ introduced a formic-acid-mediated regeneration approach for spent V_2_O_5_–WO_3_/TiO_2_ catalysts, enabling the removal of toxic arsenic (As) while preserving the catalytic activity of the catalysts. The regenerated catalysts exhibited a specific activity of 98.3% compared to fresh catalysts, achieving 99.1% As removal and less than 1.8% vanadium (V) loss within a 15 min time frame.

### 5.12 Applicability to Real Industrial Wastewater

Photocatalytic technologies have emerged as effective solutions for industrial wastewater treatment, employing advanced materials and processes to eliminate contaminants from water. The primary emphasis is on enhancing photocatalytic efficiency and devising economically viable approaches that are tailored for industrial use. Investigating metal-oxide-based semiconductors, including TiO_2_, SnO_2_, CeO_2_, ZrO_2_, WO_3_, and ZnO, researchers aim to leverage these photocatalysts in degrading organic pollutants within wastewater.^93,94^

In their 2023 study, Mei et al. investigated the challenges still preventing the widespread use of photocatalytic wastewater treatment technology in industries. They found that even though scientists have been working on photocatalysis for over three decades it is still not widely used because of various challenges. They pointed out that the efficiency of photocatalysis is not good enough, and making the treatment reactors costs a lot. To fix these problems, the researchers suggested some practical ideas, like making better photocatalysts and designing reactors that work well and can be used on a large scale.^93^

## 6 Composition of Photocatalysts

A large variety of photocatalysts (metals, metal oxides, carbon-based materials, semiconductors, quantum dots, magnetic cored dendrimer, metal–organic frameworks (MOFs), and other materials) are extensively studied for the dye degradation process in wastewater. Among the innumerable materials, we mainly focus on three generations of metal oxide for this process.

### 6.1 First-Generation Photocatalysts

**Figure 5:**
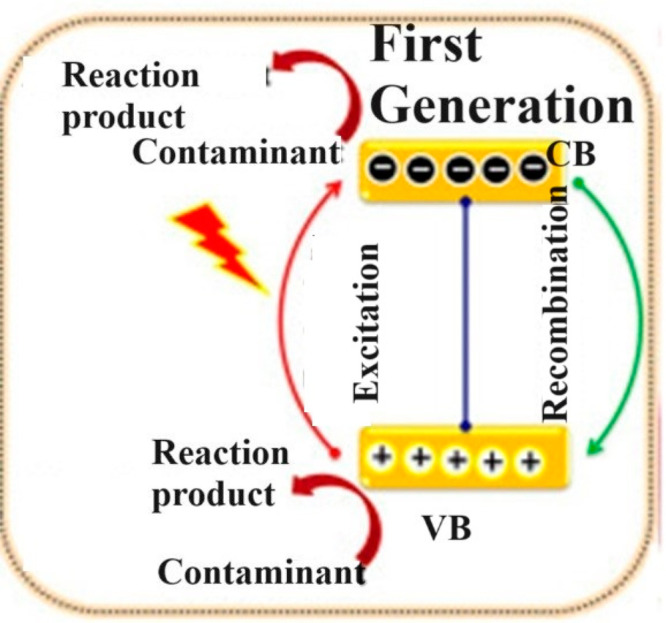
Representative schematics of the mechanism of single component metal oxide photocatalyst. Reprinted with permission from ref (102). Exclusive common copyright 2020, Elsevier.

Among the three generations, the first-generation photocatalysts (Figure 5), also known as single-component metal oxide photocatalysts, comprise TiO_2_, ZnO, ZrO_2_, ZnS, SnO_2_, CuO_2_, and NiO, etc. The operational mechanism of these photocatalysts generally depends on electron–hole pair generation under the source of UV–visible light. Among the entire single-component metal oxide photocatalysts, TiO_2_ and ZnO and particularly TiO_2_ are extensively used.

TiO_2_ mostly exists in three phases, anatase, brookite, and rutile. However, anatase is crystalline and preferably used for dye removal due to its better adsorptive affinity and higher photocatalytic affinity.^95,96^ This semiconductor acts as a good photocatalyst due to its inherent qualities like noncorrosive, nature, economical, and complete degradation of almost all the dyes in the presence of light.^97,98^

Liu et al. in 2006 synthesized TiO_2_ (anatase) by a chemical vapor deposition method for the removal of C.I. Acid Yellow17 dye. The highest dye degradation was achieved at a lower pH value and the highest intensity of light. At pH = 3, almost 70.6% dye was removed within 375 min in the presence of UV light.^58^

Gnanasekaran et al. in 2017 performed an experiment by using different metal oxides, namely, CeO_2_, CuO, NiO, Mn_3_O_4_, SnO_2_, and ZnO for the degradation of textile dyes such as methyl orange and methyl blue by using the UV–vis light. The dose of catalyst (nanoparticles) was 100 mg/100 mL solution for the experiment carried out for 2 h, and the sample was collected during the process of photocatalysis after 20 min. The results indicate that ZnO is a highly effective catalyst for the removal of methyl orange and methyl blue as compared to other metal oxides.^99^

Al Qarni et al. in 2019 worked on eco-friendly green catalyst TiO_2_ synthesized from the coffee husk extract. The structure of green nanocomposites observed by scanning electron microscopy confirms that the green TiO_2_ has macro- and micropore (8–10 nm) channels and possesses a band gap of 3.21 eV, close to the band gap of TiO_2_ obtained from ethanol. The absorbance of methylene blue by using the TiO_2_ (coffee husk extract) was rapid in the presence of irradiation (λ_max_ = 668 nm) as compared to that of TiO_2_ of ethanol. Moreover, the low cost green catalyst could be regenerated and recyclable during the reaction, and it was found that the recycled catalyst can cause 98% dye degradation from the wastewater.^100^

In 2020, Mirgane et al. described the ecofriendly catalyst ZnO synthesized from leaves of *Abelmoschus esculentus* Linn (lady finger leaves) at room temperature and pH of 9–11. The SEM and TEM analyses confirm the polycrystalline structure of ZnO. The ZnO NPs were tested for removal of methylene blue and methyl orange. The solution turns to colorless after dye degradation, and the rate of degradation increases with the passage of time (96% at 540 min). The results concluded that that this catalyst is not only eco-friendly but also very cheap and recyclable 3–4 time with good efficiency.^101^

Barakat et al. in 2011 removed Procion yellow H-EXL (commercial dye) from the wastewater by using the TiO_2_ suspension as a photocatalyst in the presence of UV light (100 W). They quantified that 100% degradation of dye was achieved by using only 1 g/L dosage of photocatalyst (TiO_2_), at pH ∼ 5 with the 10 mg/L concentration of dye.^103^

Chakarabati et al. in 2004 reported a ZnO photocatalyst for the removal of methylene blue and Eosin Y.A. from the aqueous solution in the presence of UV light (16 W). They illustrated that the dose and pH of the catalyst are directly proportional to the removal of the dyes. As the dose of catalysts is increased from 0.2 to 1 g, the % age dye degradation increased from 47 to 74% for Eosin Y.A. degraded and 58 to 76% for methylene blue during the 2 h reaction time. Meanwhile, as the pH increased from 5.5 to 9.7, the rate of degradation increased from 49 to 62%. However, in contrast as the concentration of dyes increased in the water the degradation of dyes decreased from 87% to 40% in the case of methylene blue, while Eosin Y.A. degraded from 93 to 63%.^104^

Mrunal et al. in 2019 synthesized Cu_2_O via the green synthesis approach and found that 97% methylene blue destruction occurred at pH 5.2 of the aqueous solution under the 250 W UV lamp in 120 min.^105^

Raheem et al. in 2016 utilized ZnO as a photocatalyst for the desolation of reactive green dye. They proved that 94.14% dye was destructed in the presence of 0.12 g of ZnO, while the concentration was 40 ppm.^106^

However, the first generation has certain limitations such as: (a) the catalysts of this generation, TiO_2_, ZnO, and ZrO_2_, have a large energy gap (mostly ≥3.0 eV) between CB and VB that requires only UV light for photoexcitation; (b) photocatalysts have a single VB and CB, so as the photostability increases, these electrons go back, and the process of degradation of dyes stops and in turn the efficiency of the photocatalysts decreases; and (c) these photocatalysts are used for limited dye removal, not for all dyes.

### 6.2 Second-Generation Photocatalysts

To address limitations of first-generation photocatalysts, second-generation photocatalysts were developed, featuring multicomponent combinations that significantly alter charge carrier dynamics. These photocatalysts are divided into two categories: homojunction and heterojunction photocatalysts. In homojunction photocatalysts, the semiconductor layers are composed of the same material but may have different doping levels or compositions in various regions, leading to the formation of a junction within the material itself. When these photocatalysts are exposed to light, electrons are excited from the valence band (VB) to the conduction band (CB), generating electron–hole pairs. Due to the built-in potential gradient within the semiconductor material, electrons are directed toward the region with higher CB energy, while holes migrate toward the region with lower VB energy. At the junction, electrons accumulate in the CB of the higher energy region, while holes accumulate in the VB of the lower energy region. This spatial separation of charge carriers at the junction effectively reduces recombination, thereby promoting efficient photocatalytic reactions. In contrast, heterojunction photocatalysts are designed to form junctions between different semiconductor materials, offering several advantages such as improved charge separation, enhanced catalytic activity, and broader light absorption due to varied bandgaps. In these photocatalysts, two distinct semiconductor materials are brought together, each with its own valence band (VB) and conduction band (CB) energy levels. Typically, the energy levels of one semiconductor differ from those of the other, resulting in a band offset at their interface. When illuminated, photons with energy exceeding the bandgap of the semiconductors generate electron–hole pairs within both materials. Due to the band offset, electrons migrate from the CB of one semiconductor to the CB of the other, while holes move in the opposite direction. This separation of charge carriers prevents recombination, ensuring that more electrons and holes are available for participation in photocatalytic reactions. Homojunction photocatalysts find applications in areas such as water purification, air pollution control, hydrogen production, and solar energy conversion. Heterogeneous photocatalysts are utilized in similar fields but are particularly advantageous for wastewater treatment, pollutant degradation, and environmental remediation due to their enhanced charge separation and catalytic activity at junction interfaces.^107−109^

**Figure 6:**
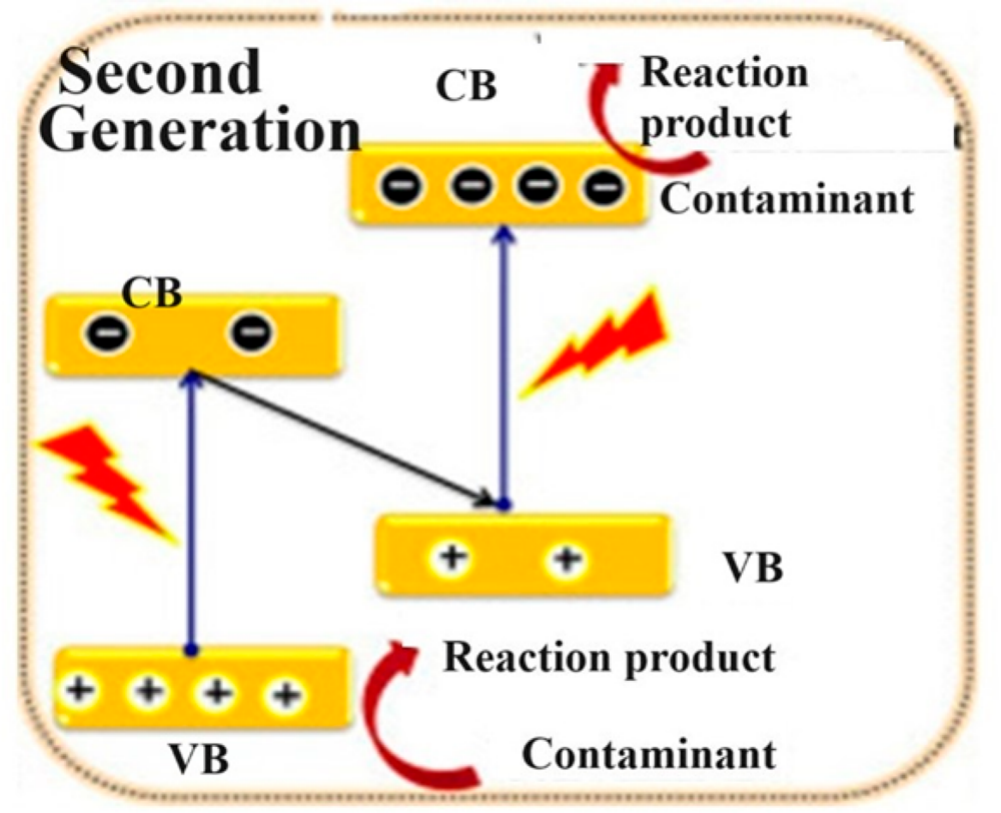
Representative schematics of the two-component heterojunction photocatalyst. Reprinted with permission from ref (102). Exclusive common copyright 2020, Elsevier.

In Figure 6, a heterogeneous photocatalytic phenomenon is illustrated wherein electrons are confined in the conduction band (CB) of one semiconductor, while holes are confined in the valence band (VB) of another semiconductor. The proposed mechanism depicts electron transfer from the CB of one semiconductor (with a high energy level) to the VB of another semiconductor (with a low energy level), or vice versa, wherein holes move from the VB of one semiconductor (with a low energy level) to the other (with a high energy level). The distinct electronic configurations lead to the generation of active sites, facilitating the degradation of dyes, achieving nearly 99% removal in a short duration. Graphitic carbon nitride (g-C_3_N_4_) and Ag_3_VO_4_ serve as exemplary instances of second-generation photocatalysts.^98^

Rodríguez León et al. in 2016 synthesized the gold nanoparticles by using natural zeolite and ascorbic acid as a reducing agent. The synthesized green nanocomposite of Au–zeolite possesses a size of 2 nm and is used for the degradation of methylene blue (MB) under LED and sunlight. The dose of catalyst was 0.15 g. The achieved degradation of methylene blue was 87% under LED light within 45 min, while the degradation of methylene blue was 100% under sunlight within 30 min. The reason for this response was that the catalyst (Au–zeolite) absorbed the oxygen into the pores of zeolite and excited the electrons on the surface of Au, which caused the formation O_2_^–^ species. As a result of the electron transfer, a partially positive charge is produced at the 5d level. The promoted electron transfer that can be neutralized by the electrons of organic molecules causes the degradation of the dyes. The results indicate that this low cost catalyst can be reused during the process and can remove the dyes 100%.^110^

Magdalene et al. in 2017 described another photocatalyst (Ce), which shows the best activity due to its shape, size, phases, and crystallographic properties. It is an environmentally friendly material, and the only limitation is that it does not produce satisfactory results alone for the degradation of dyes. So, the composite of cerium oxide with yttrium oxide (CeO_2_/Y_2_O) was synthesized by the hydrothermal process and structurally characterized by X-ray diffraction (XRD). Analysis confirms that it is a crystalline cubic face structure and has a larger amount of reactive oxygen species (ROS). For the removal of rhodamine-B (RHB), the CeO_2_/Y_2_O composite in 2:1 weight ratio was used under UV-light and as well as under visible light at different pH (3, 7, and 9). The results indicate that at different pH levels (9, 7, and 3) the degradation of dye was 98%, 95.7%, and 92.0%, respectively. When the pH was 9, the solution became colorless within 120 min; at pH 7, the solution became colorless within 180 min; and at pH 3 the degradation of dye was achieved within 180 min. The same experiment was repeated with CeO_2_/Y_2_O (2:1) at pH 9 under visible light. For the decline in absorption spectra after 553 nm with an increase of intensity of visible light, it was found that degradation of dye (RHB) was 98%, which was due to the increase in pH and surface area of the catalyst with high vicinity of oxygen which increases the efficiency of catalyst for the removal of dyes.^111^

In 2019, Zuorro et al. reported that reactive violet 5 and azo dyes are widely used in textile industries, and their presence in wastewater also increases the harmful human health effect; so, the removal of these dyes is an important issue. The nanocomposite particle Fe-doped TiO_2_ with a bandgap of 2.63 eV was utilized by them under visible light (LED) with the wavelength range of 460–470 nm, and hydrogen peroxide was added as a strong oxidizing agent. The experiment was performed by addition of 10 to 100 mM H_2_O_2_ to a dye solution at pH 10, while the concentration of dye was 30 ppm and catalyst load amount 3 g/L. The experiment reveals that due to hydrogen peroxide the reaction becomes fast and makes the solution colorless. It is due to reaction of H_2_O_2_ with TiO_2_ and production of peroxy compounds that degrade the dye more efficiently.^112^

In 2020, Hua et al. reported that C-doped TiO_2_ produces better results than TiO_2_. The basic reason is that in the case of single TiO_2_ mostly UV light has been used in various experiments, but C-doped TiO_2_ not only makes possible the transfer of light from UV to visible but also gives a better result for the removal of dyes. Hydrothermally synthesized C-doped TiO_2_ shows 90% degradation of organic pollutant under visible light in a short time due to its conductive nature.^113^

In 2021, Cruz Arias et al. reported the gold-doped TiO_2_ particle (Au-TiO_2_) for the removal of dyes. The nanocomposite of Au-TiO_2_ synthesized by using a titanium oxide precursor was tested at pH 3 for 6 h. The doping of gold was carried out by the deposition of HAuCl_4_·3H_2_O on the surface of a semiconductor. Methylene blue (MB) in different concentrations of 5, 10, 15, 20, 25, 30, 35, 40, 45, and 50 ppm was used as a model sample to check the performance of the catalyst (Au-TiO_2_) under the UV-light irradiation, and 97% dye removal was observed with an optimized dose of 30 ppm.^114^

Although the second-generation metal oxide photocatalysts are very effective and increase the % age degradation of dyes, there are some limitations such as C-doped TiO_2_ which produces improved results as compared to single TiO_2_, but the perpetration of C-doped TiO_2_ is more difficult due to the crystal lattice structure of carbon. Therefore, future research work and development is needed to make new strategies to construct the more reliable C-doped TiO_2_ with high doped amount and controlled distribution of carbon. Besides this, an extra amount of energy and cost is required for the effluent treatment to recover the second-generation photocatalyst.^113^

Saroyan et al. in 2019 illustrated that graphene oxide tapping on manganese oxide (Go-MnO) was considered a superior catalyst for the removal of organic contents due to the presence of graphene which is a strong carbonaceous material. They performed the experimental work for the removal of Reactive Black5 (RB5) in the absence of light irradiation and found that 98% of dye degraded by using only 20% manganese oxide.^115^

Ajmal et al. in 2016 designed a novel catalyst, Cu_2_O-CuO/TiO_2_, for the degradation of textile dye. They observed that under the optimal conditions almost all dyes were degraded. They reported that by using 125 mg/L of catalyst dose and at 5 mg/L dye concentration almost all the commercial dyes and specifically reactive blue 49 (RB49) were 100% degraded from the wastewater.^53^

Ma et al. in 2015 synthesized a nanocomposite photocatalyst Cu_2_O/ZnO by the coprecipitation method for the removal of orange II dye. They reported that by using the 200 mg/L dose of catalyst for the 50 mg/L initial concentration of dyes almost 80% of dye was removed from the aqueous solution.^116^

In 2020, Taddesse et al. designed a ternary metal oxide photocatalyst (Cu_2_O/ZnO/Ag_3_PO_4_) by a coprecipitation method for the removal of dye from the textile effluent. They illustrated that ternary heterogeneous metal oxide destructed 81.1% of methyl orange from the textile wastewater.^117^ Magdalane et al. in 2017 synthesized a photocatalyst CeO_2_/Y_2_O_3_ by the hydrothermal method for the degradation of dyes from urban wastewater. They observed that within 150 min under visible light at pH 9, by using 40 mg of catalyst, 94% of rhodium blue was removed from the wastewater.^118^

### 6.3 Third-Generation Photocatalysts

**Figure 7:**
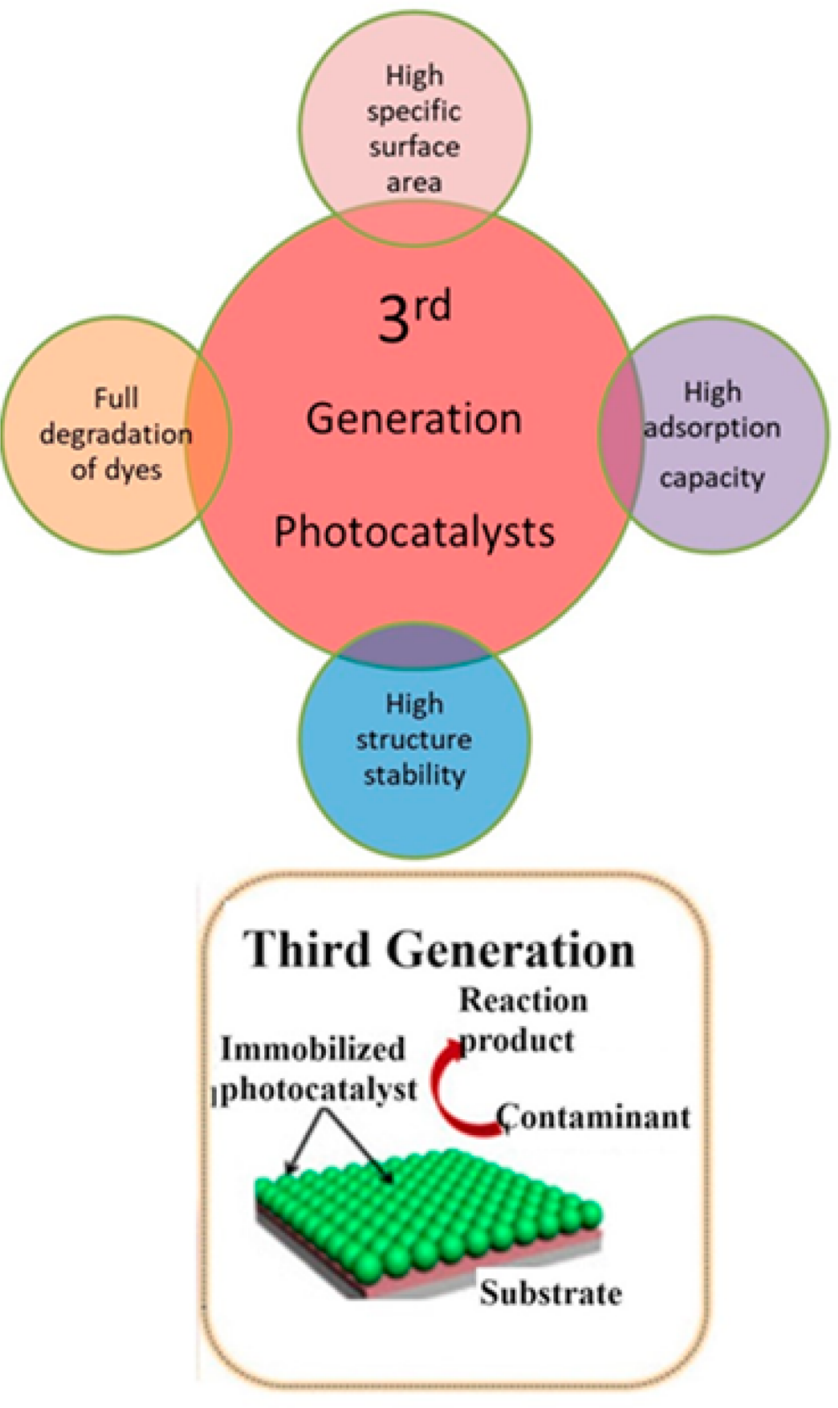
Representative schematics of the properties of three components of metal oxide (3rd generation). Reprinted in part with permission from ref (102). Exclusive common copyright 2020, Elsevier.

As in the second-generation system, there is some difficulty to remove the photocatalyst from the residue, so extra energy and cost are required for the separation; however, still all amounts of photocatalyst cannot be separated. So to avoid the separation process, the researchers reported that if a metallic photocatalyst can be attached to an organic functional group of the membrane then the separation and regeneration of photocatalysis becomes easy. So the discovery of third-generation photocatalysis gives a better solution. Third-generation photocatalysis (Figure 7) is presently attractive due to its excellent certain properties such as (i) huge surface area, (ii) high absorption tendency, (iii) complete degradation of dye, and (iv) high structure stability.^119^

Floating photocatalysts are specialized materials designed for wastewater treatment, engineered to remain suspended on the water’s surface. These photocatalysts are typically affixed to buoyant substrates such as foam or lightweight materials, allowing them to float instead of sinking. This unique design maximizes their interaction with water pollutants and exposure to sunlight, which serves as an energy source for the photocatalytic reactions. By enhancing the contact between the photocatalyst and contaminants, floating photocatalysts efficiently degrade organic pollutants and other harmful substances in wastewater.^120^ Nasir et al. note that these innovative photocatalysts utilize full solar irradiation in nonoxygenated and nonstirred reservoirs. Various fabrication techniques, such as sol–gel, surface coating, and hydrothermal methods, have been employed to synthesize these photocatalysts. Immobilizing potent photocatalysts on floatable substrates enhances their effectiveness by increasing the active surface area, optimizing light utilization, and facilitating maximum contact with pollutants. This approach holds great promise for efficient and sustainable wastewater treatment applications.^121^

In 2019, Dalponte and colleagues introduced a novel floating catalyst, TiO_2_/CaAlg, demonstrating outstanding performance under UV light with an impressive 89% decolorization rate. The researchers emphasize that this easily recoverable floating photocatalyst holds promise for enhancing the photocatalytic treatment of industrial wastewater. The design allows for efficient photoactivation without the need for mechanical stirring, offering a practical and effective solution for wastewater remediation.^122^

Khan et al. in 2012 revealed the single-step photocatalytic activity of CdS/ZnO and CdS/Al_2_O_3_ prepared using the hydrothermal process in the presence of graphene oxide (GO). During the experiment methyl orange was used as a reference dye and visible light as the source of light, and their photocatalytic activity after 10 and 60 min was observed. It was noticed that CdS/ZnO/GO and CdS/Al_2_O_3_/GO both show the highest efficiency for the degradation of organic dyes (methyl orange). However, after 10 min, the degradation efficiency of the CdS/ZnO/GO and CdS/Al_2_O_3_/GO was 85% and 51%, respectively, while after a 60 min interval, the CdS/ZnO/GO and CdS/Al_2_O_3_/GO dye degradation efficiency was 99% and 90%, respectively. The results showed that photocatalytic properties increased due to greater surface area and effective separation of photoinduced charge carriers by using the sheet like nature of GO.^123^

In 2019, Ahmed et al. synthesized a Sn-WO_3_/g-C_3_N_4_ composite in different compositions by using the calcination method and used for the removal of dyes. In all composites different amount of tin was incorporated and structure was thoroughly studied through SEM to confirm the uniform distribution of Sn-WO3 on g-sheet material. Among all composites, the 8% Sn-WO_3_/g-C_3_N(8-SnWg) structure was well ordered and more suitable for dye removal. Furthermore, the binding energy of molecular oxygen was checked by X-ray photoelectron spectroscopy (XPS) and it was found that the higher molecular oxygen adsorption on the surface of the as-prepared composite material can easily remove the organic components. The experiments performed by using 8-SnWg as a catalyst for the removal of cationic dye (Rhodamine B) and anionic dye (Methyl orange) under the visible light showed that cationic dye (Rhodamine B) removed was almost 87% in 120 min and anionic dye (Methyl orange) removal was 99% within 50 min and it proved that molecular oxygen plays an important role in the degradation of dye.^124^

Jangam et al. in 2021 synthesized a novel compound of Zn_1–*x*_Co_*x*_FeMnO_4_ with different contents of Zn with Co (*x* = 0.0, 0.25, 0.50, 0.75, 1.0) by using the sol–gel method with the addition of glycerin. The prepared samples labeled as SF-1 (*X* = 0.0), SF-2 (*X* = 0.25), SF-3 (*X* = 0.50), SF-4 (*X* = 0.75), and SF-5 (*X* = 1.0) possess bandgaps of 0.74, 2.66, 2.58, 2.24, and 2.41 eV, respectively, and these compounds were used to investigate the dye degradation efficiency under sunlight. For methylene blue (concentration 10 ppm), the observed the kinetic rate of SF-1, SF-2, SF-3, SF-4, and SF-5 was 0.063, 0.068, 0.074, 0.106, and 0.091 min^–1^, respectively. The final data prove that the rate of degradation of methylene blue was a maximum with SF-4 (0.106 min^–1^) that was attributed to the low band gap of 2.24. Further experiments carried out different metal-doped Zn ferrite nanocomposites under different sources of light to confirm that the efficiency of dye removal by the Zn_1–*x*_Co_*x*_FeMnO_4_ nanocomposite was best, as it can degrade maximum dye within 60 min under sunlight.^125^

In the same year, Anandkumar et al. introduced a novel single-phase multicomponent equiatomic oxide for the reduction of dyes under the exposure of sunlight. The nanoparticles of Gd_0.2_La_0.2_Ce_0.2_Hf_0.2_Zr_0.2_O_2_ and Gd_0.2_La_0.2_Y_0.2_Hf_0.2_Zr_0.2_O_2_ synthesized by a simple coprecipitation method at 500 °C were named as photocatalysts GLCHZ-500 and GLYHZ-500. The analysis of these compounds by XRD, UV–vis spectroscopy, and HRTEM reflects that only a single-phase cationic component was present, and their structure is in crystal lattice form. The band gap of GLCHZ-500 is 2.52 eV and of GLYHZ-500 is 3.09 eV. The experiment was performed by using these photocatalysts (GLCHZ-500, GLYHZ-500) and claimed that these semiconductors are more effective for the degradation of Cr(VI) to Cr(III) dyes, and these dyes are too toxic and cause a carcinogenic effect when dissolved in water. By using the single-phase multicomponent catalyst these carcinogenic dyes were 100% degraded, while 90% of methylene blue was degraded during the reaction.^126,127^

Bhattacharya et al. in 2019 designed a novel ternary metal oxide catalyst CuCo_0.5_Ti_0.5_O for the removal of brilliant green (BG). They demonstrated that as the catalyst loading and pH of the solution increased the dye degradation efficiency also increased. At 75g/L catalyst dosage and pH ∼ 8, above almost 90% of dye was removed from the aqueous water.^128^

Li et al. in 2008 reported Ag_2_ZnGeO_4_ with a (multiple-metal oxide) crystallite-related crystal structure with a band gap of 2.29 eV as a Vis light-sensitive photocatalyst for the process of dye degradation. The physical characterization was carried out by XRD, SEM, and UV–vis diffuse reflectance spectroscopy. The photocatalytic activity of Ag_2_ZnGeO_4_ was confirmed by using rhodamine B (RhB) and Orange II for photodegradation in an aqueous phase. After 6 h of visible light (*l* > 420 nm) exposure, the conversions of Orange II and RhB reached 69.2 and 100%, respectively. The band structure and DFT calculations reveal that the hybridized O 2p^6^ and Ag 4d^10^ orbitals develop a valence band top of Ag_2_ZnGeO_4_ and in turn narrowed the band gap as compared to the Na_2_ZnGeO_4_ parent sample. They proposed that the definite cristobalite structure of the photocatalyst favors the movement of photogenerated charge carriers and contributes positively to the experiential photocatalytic response for the dye degradation process.^129^

In Table 2, photocatalysts with a source of light which are utilized for dye degradation are summarized.

**Table 2:** Comparison of Photocatalytic Activity of Different Metal Oxides for Different Types of Dyes under Reported Conditions

Photocatalyst	Dye	Source of light	Dye concentration (mg/L)	pH	% of Dye degradation	Ref
TiO_2_	C.I. Acid yellow 17	UV	50	3	70.6	(31)
ZnO	Methyl orange	UV	16	7–8	93	(99)
TiO_2_ (CHE)	Methylene blue	Sunlight	20	-	98	(100)
ZnO	Methyl orange	UV	-	9–11	96	(101)
TiO_2_	Procion yellow H-EXL	UV	10	5	85.2	(57)
ZnO	Methylene blue	UV	50	7	76	(104)
ZnO	Eosin Y	UV	50	7	74	(104)
Cu_2_O NPs	Methylene blue	UV	0.1	5.2	97	(105)
ZnO	Reactive green	UV	40	-	94.14	(106)
Au-zeolite	Methyl orange	Sunlight	15	-	100	(110)
CeO_2_/Y_2_O	Rhodamine B	Visible		9	98	(111)
Fe-doped TiO_2_	Reactive violet 5	Visible	3	9	87.2	(112)
C-doped titania	Methylene blue	UV	-	-	90	(113)
Au-TiO_2_	Methylene blue	UV	30	3	97.4	(114)
GO-MnO_2_	Reactive black 5	UV	60	3	85	(115)
4 wt % Cu_2_O-CuO/TiO_2_	Reactive Blue 49	UV	5	6.5	100	(53)
Cu_2_O/ZnO	Orange II	Visible	50	6.4	80	(116)
Cu_2_O/ZnO/Ag_3_PO_4_	Methyl orange	Visible	10	6	81	(117)
CeO_2_/Y_2_O_3_	Rhodamine B	UV–vis	40	9	98	(118)
CdS/ZnO/GO	Methylene blue	UV	-	-	99	(123)
Sn-WO_3_/g-C_3_N_4_	Rhodamine B	UV–vis	10		99	(124)
Zn_0.25_Co_0.75_ MnFeO_4_	Methylene blue	UV–vis	-	2.5	99	(125)
GLCHZ-500	Methylene blue	Sunlight	10	-	75	(127)
CuCo_0.5_Ti_0.5_O_2_	Brilliant green	Visible	5	8	95	(128)
Ag_2_ZnGeO_4_	Orange II	Visible	12		69.2	(129)
Ag_2_ZnGeO_4_	Rhodium blue	Visible	12	-	100	(129)

## 7 Conclusion and Future Prospective

Dye removal from wastewater is imperative to preventing detrimental impacts on ecosystems and human health. Various techniques are employed for wastewater treatment, including biological treatment, physiochemical treatment, membrane filtering, advanced oxidation processes (AOPs), and photocatalytic treatment. However, conventional methods have limitations such as an inability to remove a wide range of impurities, reliance on external chemicals, production of slurry and sludge, and the need for routine maintenance. In contrast, the photocatalytic treatment of wastewater offers several advantages. It results in the complete breakdown of pollutants into simpler compounds without the use of external chemicals. Additionally, photocatalysts can be regenerated and reused for further processing. Photocatalysis has shown significant potential for decomposing dyes, hydrocarbons, insecticides, germs, and microorganisms and reducing dangerous metal ions in wastewater. During photocatalysis, up to 80–90% of pigments can be removed from wastewater without the use of chemicals. However, the performance of photocatalysts depends on various factors such as catalytic morphology, electron–hole pair generation and recombination rate, pH, temperature, and light intensity, among others. Optimization of these parameters is crucial to achieving better results.

In this review, three generations of metal oxides are thoroughly studied. The first generation has been widely used, while its limitations in degrading all dyes and reliance on UV light have prompted the exploration of second- and third-generation metal oxides. These newer generations exhibit better charge separation, faster reaction kinetics, utilization of visible light, and improved dye degradation tendencies. Additionally, immobilization technology in third-generation photocatalysts offers opportunities for catalyst regeneration. The shape and surface area of the catalyst also influence dye removal, with larger surface areas enhancing the removal efficiency.

Despite that photocatalysis is an advanced technology for wastewater treatment and especially for dye degradation, work is still required to enhance the efficiency of the process. So there are several future recommendations.

- Further research is required to focus on maximizing the utilization of natural sunlight in photocatalysis processes to enhance the economic feasibility of the treatment.
- There is a need for in-depth investigation into the mechanism of photocatalysis at a molecular level to gain a comprehensive understanding of the process. Understanding the formation and transformation of intermediates will enable the optimization of photocatalytic degradation pathways and improve overall process efficiency.
- Additional research is needed to optimize the design of photocatalysis reactors by enhancing catalyst efficiency, integrating mathematical modeling for process optimization, and implementing automated control systems to enhance operational efficiency and overall effectiveness.

## References

[ref1] Al-Ghouti M. A.; et al. The removal of dyes from textile wastewater: a study of the physical characteristics and adsorption mechanisms of diatomaceous earth. Journal of Environmental Management 2003, 69 (3), 229–238. 10.1016/j.jenvman.2003.09.005. 14580724

[ref2] Alinsafi A.; et al. Electro-coagulation of reactive textile dyes and textile wastewater. Chemical Engineering and Processing: Process Intensification 2005, 44 (4), 461–470. 10.1016/j.cep.2004.06.010.

[ref3] Anliker R. Ecotoxicology of dyestuffs—A joint effort by industry. Ecotoxicology and Environmental Safety 1979, 3 (1), 59–74. 10.1016/0147-6513(79)90060-5. 540549

[ref4] Quansah J. O.; et al. Nascent rice husk as an adsorbent for removing cationic dyes from textile wastewater. Applied Sciences 2020, 10 (10), 3437 10.3390/app10103437.

[ref5] AbdurRahman F. B.; Akter M.; Abedin M. Z. Dyes removal from textile wastewater using orange peels. Int. J. Sci. Technol. Res. 2013, 2 (9), 47–50. 10.3390/w14244104.

[ref6] Harrelkas F.; et al. Photocatalytic and combined anaerobic–photocatalytic treatment of textile dyes. Chemosphere 2008, 72 (11), 1816–1822. 10.1016/j.chemosphere.2008.05.026. 18585754

[ref7] Prier C. K.; Rankic D.A.; MacMillan D.W.J.C.r. Visible light photoredox catalysis with transition metal complexes: applications in organic synthesis. Chem. Rev. 2013, 113 (7), 5322–5363. 10.1021/cr300503r. 23509883 PMC4028850

[ref8] Kim H.-i.; et al. Enhanced photocatalytic and photoelectrochemical activity in the ternary hybrid of CdS/TiO2/WO3 through the cascadal electron transfer. J. Phys. Chem. C 2011, 115 (19), 9797–9805. 10.1021/jp1122823.

[ref9] Gaigneaux E., Scientific Bases for the Preparation of Heterogeneous Catalysts: Proceedings of the 10th International Symposium, Louvain-la-Neuve, Belgium; Elsevier, 2010. 10.1007/s10562-010-0541-7.

[ref10] Bhatkhande D. S.; Pangarkar V. G.; Beenackers A. A. C. M. Photocatalytic degradation for environmental applications–a review. Journal of Chemical Technology & Biotechnology: International Research in Process, Environmental & Clean Technology 2002, 77 (1), 102–116. 10.1002/jctb.532.

[ref11] Gaya U. I.; Abdullah A. H. Heterogeneous photocatalytic degradation of organic contaminants over titanium dioxide: a review of fundamentals, progress and problems. Journal of photochemistry and photobiology C: Photochemistry reviews 2008, 9 (1), 1–12. 10.1016/j.jphotochemrev.2007.12.003.

[ref12] Malato S. Decontamination and disinfection of water by solar photocatalysis: recent overview and trends. Catalysis Today 2009, 147 (1), 1–59. 10.1016/j.cattod.2009.06.018.

[ref13] Gregory P. Classification of dyes by chemical structure. In The chemistry and application of dyes; Springer; 1990, pp 17–47. 10.1007/978-1-4684-7715-3_2.

[ref14] Panda S. K.; et al. Magnetite nanoparticles as sorbents for dye removal: a review. Environmental Chemistry Letters 2021, 19 (3), 2487–2525. 10.1007/s10311-020-01173-9.

[ref15] Mukerjee P. J. A. C. Use of ionic dyes in analysis of ionic surfactants and other ionic organic compounds. Anal. Chem. 1956, 28 (5), 870–873. 10.1021/ac60113a026.

[ref16] Faria P. C. C.; Órfão J. J. M.; Pereira M. F. R. Adsorption of anionic and cationic dyes on activated carbons with different surface chemistries. Water Res. 2004, 38 (8), 2043–2052. 10.1016/j.watres.2004.01.034. 15087185

[ref17] Rauf M. A.; Ashraf S. S. Fundamental principles and application of heterogeneous photocatalytic degradation of dyes in solution. Chem. Eng. J. 2009, 151 (1), 10–18. 10.1016/j.cej.2009.02.026.

[ref18] Gürses A., Classification of dye and pigments. In Dyes and pigments; Springer, 2016; pp 31–45. 10.1007/978-3-319-33892-7_3.

[ref19] El Harfi S.; El Harfi A. Classifications, properties and applications of textile dyes: A review. Applied Journal of Environmental Engineering Science 2017, 3 (3), 311–320. 10.48422/IMIST.PRSM/ajees-v3i3.9681.

[ref20] Uddin F. J. C. Environmental hazard in textile dyeing wastewater from local textile industry. Cellulose 2021, 28 (17), 10715–10739. 10.1007/s10570-021-04228-4.

[ref21] Teng T. T.; Low L.W. Removal of dyes and pigments from industrial effluents. In Advances in Water Treatment and Pollution Prevention; Springer, 2012; pp 65–93. 10.1007/978-94-007-4204-8_4.

[ref22] Kadhom M.; et al. Removal of dyes by agricultural waste. Sustainable Chemistry and Pharmacy 2020, 16, 100259 10.1016/j.scp.2020.100259.

[ref23] Jain A. K.; et al. Utilization of industrial waste products as adsorbents for the removal of dyes. J. Hazard. Mater. 2003, 101 (1), 31–42. 10.1016/S0304-3894(03)00146-8. 12850318

[ref24] Liu H.; et al. Biosynthesis based membrane filtration coupled with iron nanoparticles reduction process in removal of dyes. Chem. Eng. J. 2020, 387, 124202 10.1016/j.cej.2020.124202.

[ref25] Jankowska K.; et al. Synergistic action of laccase treatment and membrane filtration during removal of azo dyes in an enzymatic membrane reactor upgraded with electrospun fibers. J. Hazard. Mater. 2022, 435, 129071 10.1016/j.jhazmat.2022.129071. 35650748

[ref26] Hassan M. M.; Carr C. M. A critical review on recent advancements of the removal of reactive dyes from dyehouse effluent by ion-exchange adsorbents. Chemosphere 2018, 209, 201–219. 10.1016/j.chemosphere.2018.06.043. 29933158

[ref27] Selvam K.; Swaminathan K.; Chae K.-S. Decolourization of azo dyes and a dye industry effluent by a white rot fungus Thelephora sp. Bioresour. Technol. 2003, 88 (2), 115–119. 10.1016/S0960-8524(02)00280-8. 12576004

[ref28] Jun L. Y.; et al. An overview of immobilized enzyme technologies for dye and phenolic removal from wastewater. Journal of Environmental Chemical Engineering 2019, 7 (2), 102961 10.1016/j.jece.2019.102961.

[ref29] Arslan I.; Balcioğlu I. A. Degradation of commercial reactive dyestuffs by heterogenous and homogenous advanced oxidation processes: a comparative study. Dyes Pigm. 1999, 43 (2), 95–108. 10.1016/S0143-7208(99)00048-0.

[ref30] Wijannarong S.; et al. Removal of Reactive Dyes from Textile Dyeing Industrial Effluent by Ozonation Process. APCBEE Procedia 2013, 5, 279–282. 10.1016/j.apcbee.2013.05.048.

[ref31] Chen X.; et al. Preparation of ZnO photocatalyst for the efficient and rapid photocatalytic degradation of azo dyes. Nanoscale Res. Lett. 2017, 12 (1), 1–10. 10.1186/s11671-017-1904-4. 28235375 PMC5319938

[ref32] Yi F.; Chen S.; Yuan C.e. Effect of activated carbon fiber anode structure and electrolysis conditions on electrochemical degradation of dye wastewater. J. Hazard. Mater. 2008, 157 (1), 79–87. 10.1016/j.jhazmat.2007.12.093. 18258359

[ref33] Reza K. M.; Kurny A.; Gulshan F. Parameters affecting the photocatalytic degradation of dyes using TiO 2: a review. Applied Water Science 2017, 7 (4), 1569–1578. 10.1007/s13201-015-0367-y.

[ref34] Singh P.; et al. Systematic review on applicability of magnetic iron oxides–integrated photocatalysts for degradation of organic pollutants in water. Materials Today Chemistry 2019, 14, 100186 10.1016/j.mtchem.2019.08.005.

[ref35] Jamee R.; Siddique R. Biodegradation of synthetic dyes of textile effluent by microorganisms: an environmentally and economically sustainable approach. European journal of microbiology and immunology 2019, 9 (4), 114–118. 10.1556/1886.2019.00018. 31934362 PMC6945995

[ref36] Bhatnagar A.; et al. An overview of the modification methods of activated carbon for its water treatment applications. Chem. Eng. J. 2013, 219, 499–511. 10.1016/j.cej.2012.12.038.

[ref37] Huang C.-P. Recent Advances in Advanced Oxidation Processes. In Proceedings of the Korean Environmental Sciences Society Conference; The Korean Environmental Sciences Society, 1998. 10.1021/ie00095a001.

[ref38] Ghernaout D.; Elboughdiri N.J.O.A.L.J. Advanced oxidation processes for wastewater treatment: facts and future trends. OALib 2020, 7 (2), 1–15. 10.4236/oalib.1106139.

[ref39] Litter M.; Quici N.J.R.P.o.E. Photochemical advanced oxidation processes for water and wastewater treatment. ENG 2010, 4 (3), 217–241. 10.2174/187221210794578574.

[ref40] Sinha S.; Nigam S.; Syed M. Insight into Advanced Oxidation Processes for Wastewater Treatment. In Advanced Oxidation Processes for Wastewater Treatment; CRC Press, 2022; pp 101–106. 10.1201/9781003165958.

[ref41] Viswanathan B. Photocatalytic degradation of dyes: An overview. Current Catalysis 2018, 7 (2), 99–121. 10.2174/2211544707666171219161846.

[ref42] Hoffmann M. R. Environmental applications of semiconductor photocatalysis. Chem. Rev. 1995, 95 (1), 69–96. 10.1021/cr00033a004.

[ref43] Zhang H.; Chen G.; Bahnemann D.W. Photoelectrocatalytic materials for environmental applications. J. Mater. Chem. 2009, 19 (29), 5089–5121. 10.1039/b821991e.

[ref44] Becker C. From Langmuir to Ertl: The “Nobel” History of the Surface Science Approach to Heterogeneous Catalysis; Elsevier, 2018. 10.1016/B978-0-12-409547-2.13527-9.

[ref45] Rauf M.; Ashraf S. S. Fundamental principles and application of heterogeneous photocatalytic degradation of dyes in solution. Chemical engineering journal 2009, 151 (1–3), 10–18. 10.1016/j.cej.2009.02.026.

[ref46] Rao A. N.; Sivasankar B.; Sadasivam V. Kinetic studies on the photocatalytic degradation of Direct Yellow 12 in the presence of ZnO catalyst. J. Mol. Catal. A: Chem. 2009, 306 (1–2), 77–81. 10.1016/j.molcata.2009.02.028.

[ref47] Masarwa A.; et al. Oxidation of organic substrates in aerated aqueous solutions by the Fenton reagent. Coord. Chem. Rev. 2005, 249 (17–18), 1937–1943. 10.1016/j.ccr.2005.01.003.

[ref48] Chamarro E.; Marco A.; Esplugas S. Use of Fenton reagent to improve organic chemical biodegradability. Water research 2001, 35 (4), 1047–1051. 10.1016/S0043-1354(00)00342-0. 11235870

[ref49] Koppenol W. J. P. Names for inorganic radicals (IUPAC recommendations 2000). A. Chemistry 2000, 72 (3), 437–446. 10.1351/pac200072030437.

[ref50] Braslavsky S. E. Glossary of terms used in photocatalysis and radiation catalysis (IUPAC Recommendations 2011). Pure Appl. Chem. 2011, 83 (4), 931–1014. 10.1351/PAC-REC-09-09-36.

[ref51] Torres R.; et al. A comparative study of ultrasonic cavitation and Fenton’s reagent for bisphenol A degradation in deionised and natural waters. Journal of hazardous materials 2007, 146 (3), 546–551. 10.1016/j.jhazmat.2007.04.056. 17532122

[ref52] Nazri M. K. H. M.; Sapawe N. A short review on photocatalytic toward dye degradation. Materials Today: Proceedings 2020, 31, A42–A47. 10.1016/j.matpr.2020.10.967.

[ref53] Ajmal A.; et al. Photocatalytic degradation of textile dyes on Cu2O-CuO/TiO2 anatase powders. Journal of Environmental Chemical Engineering 2016, 4 (2), 2138–2146. 10.1016/j.jece.2016.03.041.

[ref54] Will I.; et al. Photo-Fenton degradation of wastewater containing organic compounds in solar reactors. Sep. Purif. Technol. 2004, 34 (1–3), 51–57. 10.1016/S1383-5866(03)00174-6.

[ref55] Habibi M. H.; Vosooghian H. Photocatalytic degradation of some organic sulfides as environmental pollutants using titanium dioxide suspension. J. Photochem. Photobiol., A 2005, 174 (1), 45–52. 10.1016/j.jphotochem.2005.02.012.

[ref56] Rauf M.; Ansari F.; Abbasi G. Photocatalytic degradation of some azodyes. Magallat kulliyyat al-ulum. Gamiat imarat al-arabiyyat al-muttahidat 2004, 13 (1425H), 41–45. 10.1016/j.jtice.2022.104663.

[ref57] Malik P.; Saha S. Oxidation of direct dyes with hydrogen peroxide using ferrous ion as catalyst. Sep. Purif. Technol. 2003, 31 (3), 241–250. 10.1016/S1383-5866(02)00200-9.

[ref58] Liu C.-C.; et al. Photodegradation treatment of azo dye wastewater by UV/TiO2 process. Dyes Pigm. 2006, 68 (2–3), 191–195. 10.1016/j.dyepig.2004.12.002.

[ref59] Alnuaimi M. M.; Rauf M.; Ashraf S. S. Comparative decoloration study of Neutral Red by different oxidative processes. Dyes Pigm. 2007, 72 (3), 367–371. 10.1016/j.dyepig.2005.09.020.

[ref60] Singh P.; et al. Photocatalytic degradation of Acid Red dye stuff in the presence of activated carbon-TiO2 composite and its kinetic enumeration. Journal of Water Process Engineering 2016, 12, 20–31. 10.1016/j.jwpe.2016.04.007.

[ref61] Rauf M.; Meetani M.; Hisaindee S. An overview on the photocatalytic degradation of azo dyes in the presence of TiO2 doped with selective transition metals. Desalination 2011, 276 (1–3), 13–27. 10.1016/j.desal.2011.03.071.

[ref62] Gaya U. I.; et al. Photocatalytic treatment of 4-chlorophenol in aqueous ZnO suspensions: Intermediates, influence of dosage and inorganic anions. Journal of hazardous materials 2009, 168 (1), 57–63. 10.1016/j.jhazmat.2009.01.130. 19268454

[ref63] Chen C.; et al. UV light induced photodegradation of malachite green on TiO2 nanoparticles. Journal of hazardous materials 2007, 141 (3), 520–528. 10.1016/j.jhazmat.2006.07.011. 16934397

[ref64] Chin P.; Yang L. P.; Ollis D. F. Formaldehyde removal from air via a rotating adsorbent combined with a photocatalyst reactor: Kinetic modeling. J. Catal. 2006, 237 (1), 29–37. 10.1016/j.jcat.2005.10.013.

[ref65] Hassan S. S.; et al. Green synthesis and characterization of ZnO nanoparticles for photocatalytic degradation of anthracene. Advances in Natural Sciences: Nanoscience and Nanotechnology 2015, 6 (4), 045012 10.1088/2043-6262/6/4/045012.

[ref66] Tan R.; et al. Reducing competition by coordinating solvent promotes morphological control in alternating layer growth of CdSe/CdS core/shell quantum dots. Chem. Mater. 2015, 27 (21), 7468–7480. 10.1021/acs.chemmater.5b03588.

[ref67] Xie Z.; et al. Enhanced photoelectrochemical properties of TiO2 nanorod arrays decorated with CdS nanoparticles. Sci. Technol. Adv. Mater. 2014, 15, 055006 10.1088/1468-6996/15/5/055006. 27877718 PMC5099679

[ref68] Elaziouti A.; Ahmed B. ZnO-assisted photocatalytic degradation of congo Red and benzopurpurine 4B in aqueous solution. J. Chem. Eng. Process Technol. 2011, 2, 1–9. 10.4172/2157-7048.1000106.

[ref69] Yoon T. P.; Ischay M.A.; Du J.J.N.c. Visible light photocatalysis as a greener approach to photochemical synthesis. Nature Chem 2010, 2 (7), 527–532. 10.1038/nchem.687. 20571569

[ref70] Mugumo R. Visible-Light-Induced Photocatalytic Degradation of Rhodamine B Dye Using CuS/ZnS PN Heterojunction Nanocomposite under Visible Light Irradiation. Catalysts 2023, 13, 1184 10.3390/catal13081184.

[ref71] Alwared A. I. Solar-Induced Photocatalytic Degradation of Reactive Red and Turquoise Dyes Using a Titanium Oxide/Xanthan Gum Composite. Sustainability 2023, 15 (14), 10815 10.3390/su151410815.

[ref72] Karuppaiya V. Preparation of fluorinated zirconia doped with tin oxide nanocomposites for photocatalytic degradation of organic dyes in contaminated water bodies. Zeitschrift für Physikalische Chemie 2024, 10.1515/zpch-2023-0501.

[ref73] Hosseini-Zori M.; Shourijeh Z. M. Synthesis, characterization and investigation of photocatalytic activity of transition metal-doped TiO2 nanostructures. Progress in Color, Colorants and Coatings 2018, 11 (4), 209–220. 10.30509/pccc.2018.76671.

[ref74] Anwer H.; Park J.-W. Synthesis and characterization of a heterojunction rGO/ZrO2/Ag3PO4 nanocomposite for degradation of organic contaminants. Journal of hazardous materials 2018, 358, 416–426. 10.1016/j.jhazmat.2018.07.019. 30007252

[ref75] Tahir K.; et al. An efficient photo catalytic activity of green synthesized silver nanoparticles using Salvadora persica stem extract. Sep. Purif. Technol. 2015, 150, 316–324. 10.1016/j.seppur.2015.07.012.

[ref76] Ajmal A.; et al. Principles and mechanisms of photocatalytic dye degradation on TiO 2 based photocatalysts: a comparative overview. Rsc Advances 2014, 4 (70), 37003–37026. 10.1039/C4RA06658H.

[ref77] Kashif N.; Ouyang F. Parameters effect on heterogeneous photocatalysed degradation of phenol in aqueous dispersion of TiO2. Journal of Environmental Sciences 2009, 21 (4), 527–533. 10.1016/S1001-0742(08)62303-7. 19634430

[ref78] He L.; et al. Experimental studies on magnetization in the excited state by using the magnetic field effect of light scattering based on multi-layer graphene particles suspended in organic solvents. Nanoscale 2017, 9 (7), 2563–2568. 10.1039/C6NR08148G. 28150824

[ref79] Mena E.; et al. Reaction mechanism and kinetics of DEET visible light assisted photocatalytic ozonation with WO3 catalyst. Appl. Catal. B: Environmental 2017, 202, 460–472. 10.1016/j.apcatb.2016.09.029.

[ref80] Bui T. D.; et al. Lowering of photocatalytic activity of TiO2 particles during oxidative decomposition of benzene in aerated liquid. Appl. Catal. B: Environmental 2010, 94 (1–2), 186–191. 10.1016/j.apcatb.2009.11.008.

[ref81] Guillard C.; et al. Influence of chemical structure of dyes, of pH and of inorganic salts on their photocatalytic degradation by TiO2 comparison of the efficiency of powder and supported TiO2. J. Photochem. Photobiol., A 2003, 158 (1), 27–36. 10.1016/S1010-6030(03)00016-9.

[ref82] Hu C.; et al. Effects of acidity and inorganic ions on the photocatalytic degradation of different azo dyes. Appl. Catal. B: Environmental 2003, 46 (1), 35–47. 10.1016/S0926-3373(03)00139-5.

[ref83] Steky F. V. Contribution of the lamellar morphology to the photocatalytic activity of alkaline-hydrothermally treated titania in rhodamine B photodegradation. Phys. Chem. Chem. Phys. 2023, 25 (6), 5183–5195. 10.1039/D2CP05098F. 36723401

[ref84] Shenoy M. R. The effect of morphology-dependent surface charges of iron oxide on the visible light photocatalytic degradation of methylene blue dye. J Mater Sci: Mater Electron 2020, 31, 17703–17717. 10.1007/s10854-020-04325-3.

[ref85] Tuc Altaf C. Impact on the Photocatalytic Dye Degradation of Morphology and Annealing-Induced Defects in Zinc Oxide Nanostructures. ACS Omega 2023, 8 (17), 14952–14964. 10.1021/acsomega.2c07412. 37151495 PMC10157689

[ref86] Das B. Morphology tuned Ga2O3 nanostructures for visible light-assisted dye-sensitized photocatalytic water remediation. Materials Today Communications 2023, 10.1016/j.mtcomm.2023.105849.

[ref87] Weng B. Photocorrosion inhibition of semiconductor-based photocatalysts: basic principle, current development, and future perspective. ACS Catal. 2019, 9 (5), 4642–4687. 10.1021/acscatal.9b00313.

[ref88] Warren Z.; Wenk J.; Mattia D.J.R.a. Increased photocorrosion resistance of ZnO foams via transition metal doping. RSC Adv. 2023, 13 (4), 2438–2450. 10.1039/D2RA06730G. 36741143 PMC9844254

[ref89] Miranda-García N. Regeneration approaches for TiO2 immobilized photocatalyst used in the elimination of emerging contaminants in water. Catalysis Today 2014, 230, 27–34. 10.1016/j.cattod.2013.12.048.

[ref90] Liao W. Photocatalyst immobilized by hydrogel, efficient degradation and self regeneration: A review. Materials Science in Semiconductor Processing 2022, 10.1016/j.mssp.2022.106929.

[ref91] Miranda-García N.; et al. Regeneration approaches for TiO2 immobilized photocatalyst used in the elimination of emerging contaminants in water. Catal. Today 2014, 230, 27–34. 10.1016/j.cattod.2013.12.048.

[ref92] Kim T. Photo-Regeneration of Zeolite-Based Volatile Organic Compound Filters Enabled by TiO2 Photocatalyst. Nanomaterials 2022, 12 (17), 2959 10.3390/nano12172959. 36079996 PMC9457909

[ref93] Yin Y.; Li X.; Li K.; Liu R.; Wu H.; Zhu T. Formic Acid-Mediated Regeneration Strategy for As-Poisoned V _2_ O _5_ -WO _3_ /TiO _2_ Catalysts with Lossless Catalytic Activity and Simultaneous As Recycling. Environ. Sci. Technol. 2022, 56 (17), 12625–12634. 10.1021/acs.est.2c04613. 35947769

[ref94] Datta P.; Roy S. J. C. R. Recent Development of Photocatalytic Application Towards Wastewater Treatment. Catal. Res. 2023, 3 (3), 1–23. 10.21926/cr.2303020.

[ref95] Jolivet J.-P.; et al. Design of metal oxide nanoparticles: Control of size, shape, crystalline structure and functionalization by aqueous chemistry. Comptes Rendus Chimie 2010, 13 (1–2), 40–51. 10.1016/j.crci.2009.09.012.

[ref96] Ali M.; et al. A review on the recent developments in zirconium and carbon-based catalysts for photoelectrochemical water-splitting. Int. J. Hydrogen Energy 2021, 46 (35), 18257–18283. 10.1016/j.ijhydene.2021.02.202.

[ref97] Ali M.; et al. A review on the recent developments in zirconium and carbon-based catalysts for photoelectrochemical water-splitting. Int. J. Hydrogen Energy 2021, 46, 18257 10.1016/j.ijhydene.2021.02.202.

[ref98] Anwer H.; et al. Photocatalysts for degradation of dyes in industrial effluents: Opportunities and challenges. Nano Research 2019, 12 (5), 955–972. 10.1007/s12274-019-2287-0.

[ref99] Gnanasekaran L.; et al. Synthesis and characterization of metal oxides (CeO2, CuO, NiO, Mn3O4, SnO2 and ZnO) nanoparticles as photo catalysts for degradation of textile dyes. Journal of Photochemistry and Photobiology B: Biology 2017, 173, 43–49. 10.1016/j.jphotobiol.2017.05.027. 28558305

[ref100] Al Qarni F.; Alomair N. A.; Mohamed H. H. Environment-friendly nanoporous titanium dioxide with enhanced photocatalytic activity. Catalysts 2019, 9 (10), 799 10.3390/catal9100799.

[ref101] Mirgane N. A.; et al. Degradation of dyes using biologically synthesized zinc oxide nanoparticles. Materials Today: Proceedings 2021, 37, 849–853. 10.1016/j.matpr.2020.06.037.

[ref102] Olatunde O. C.; Kuvarega A. T.; Onwudiwe D. C. Photo enhanced degradation of contaminants of emerging concern in waste water. Emerging Contaminants 2020, 6, 283–302. 10.1016/j.emcon.2020.07.006.

[ref103] Barakat M. A. Adsorption and photodegradation of Procion yellow H-EXL dye in textile wastewater over TiO2 suspension. Journal of Hydro-environment Research 2011, 5 (2), 137–142. 10.1016/j.jher.2010.03.002.

[ref104] Chakrabarti S.; Dutta B. K. Photocatalytic degradation of model textile dyes in wastewater using ZnO as semiconductor catalyst. J. Hazard. Mater. 2004, 112 (3), 269–278. 10.1016/j.jhazmat.2004.05.013. 15302448

[ref105] Mrunal V. K.; Vishnu A. K.; Momin N.; Manjanna J. Cu2O nanoparticles for adsorption and photocatalytic degradation of methylene blue dye from aqueous medium. Environmental Nanotechnology, Monitoring & Management 2019, 12, 100265 10.1016/j.enmm.2019.100265.

[ref106] Raheem R. A.; Al-gubury H. Y.; Aljeboree A. M.; Alkaim A. F. Photocatalytic degradation of reactive green dye by using Zinc oxide. Journal of Chemical and Pharmaceutical Sciences 2016, 9 (3), 1134–1138. 10.30558/jchps.2016.

[ref107] Phang S. J.; et al. Recent advances in homojunction-based photocatalysis for sustainable environmental remediation and clean energy generation. Appl. Mater. Today 2020, 20, 100741 10.1016/j.apmt.2020.100741.

[ref108] Habibi-Yangjeh A.; Pournemati K. A review on emerging homojunction photocatalysts with impressive performances for wastewater detoxification. Critical Reviews in Environmental Science and Technology 2024, 54 (4), 290–320. 10.1080/10643389.2023.2239125.

[ref109] Asadi A. M. S. Catalysts for advanced oxidation processes: Deep eutectic solvents-assisted synthesis–A review. Water Resources and Industry 2024, 10.1016/j.wri.2024.100251.

[ref110] León E. R.; et al. Study of methylene blue degradation by gold nanoparticles synthesized within natural zeolites. J. Nanomater. 2016, 2016, 1 10.1155/2016/9541683.

[ref111] Magdalane C. M.; et al. Evaluation on the heterostructured CeO2/Y2O3 binary metal oxide nanocomposites for UV/Vis light induced photocatalytic degradation of Rhodamine-B dye for textile engineering application. J. Alloys Compd. 2017, 727, 1324–1337. 10.1016/j.jallcom.2017.08.209.

[ref112] Zuorro A.; et al. Photocatalytic degradation of azo dye reactive violet 5 on Fe-doped titania catalysts under visible light irradiation. Catalysts 2019, 9 (8), 645 10.3390/catal9080645.

[ref113] Hua L.; Yin Z.; Cao S. Recent advances in synthesis and applications of carbon-doped TiO2 nanomaterials. Catalysts 2020, 10 (12), 1431 10.3390/catal10121431.

[ref114] Arias M.-C.; et al. Removal of the Methylene Blue Dye (MB) with Catalysts of Au-TiO 2: Kinetic and Degradation Pathway. Modern Research in Catalysis 2021, 10 (01), 1 10.4236/mrc.2021.101001.

[ref115] Saroyan H.; Kyzas G. Z.; Deliyanni E. A. Effective dye degradation by graphene oxide supported manganese oxide. Processes 2019, 7 (1), 40 10.3390/pr7010040.

[ref116] Ma J.; et al. Visible-light photocatalytic decolorization of Orange II on Cu2O/ZnO nanocomposites. Ceram. Int. 2015, 41, 2050–2056. 10.1016/j.ceramint.2014.09.137.

[ref117] Taddesse A. M.; Alemu M.; Kebede T. Enhanced photocatalytic activity of p-n-n heterojunctions ternary composite Cu2O/ZnO/Ag3PO4 under visible light irradiation. Journal of Environmental Chemical Engineering 2020, 8 (5), 104356 10.1016/j.jece.2020.104356.

[ref118] Magdalane C. M.; et al. Evaluation on the heterostructured CeO2/Y2O3 binary metal oxide nanocomposites for UV/Vis light induced photocatalytic degradation of Rhodamine - B dye for textile engineering application. J. Alloys Compd. 2017, 727, 1324–1337. 10.1016/j.jallcom.2017.08.209.

[ref119] Jiang W.; et al. Three-dimensional photocatalysts with a network structure. Journal of Materials Chemistry A 2017, 5 (12), 5661–5679. 10.1039/C7TA00398F.

[ref120] Xing Z. Recent advances in floating TiO2-based photocatalysts for environmental application. Applied Catalysis B: Environmental 2018, 225, 452–467. 10.1016/j.apcatb.2017.12.005.

[ref121] Nasir A. M.; et al. A review on floating nanocomposite photocatalyst: Fabrication and applications for wastewater treatment. Journal of Water Process Engineering 2020, 36, 101300 10.1016/j.jwpe.2020.101300.

[ref122] Dalponte I.; et al. Formulation and optimization of a novel TiO2/calcium alginate floating photocatalyst. Int. J. Biol. Macromol. 2019, 137, 992–1001. 10.1016/j.ijbiomac.2019.07.020. 31279883

[ref123] Khan Z.; et al. Visible light assisted photocatalytic hydrogen generation and organic dye degradation by CdS–metal oxide hybrids in presence of graphene oxide. RSC Adv. 2012, 2 (32), 12122–12128. 10.1039/c2ra21596a.

[ref124] Ahmed K. E.; et al. Synthesis of Sn-WO3/g-C3N4 composites with surface activated oxygen for visible light degradation of dyes. J. Photochem. Photobiol., A 2019, 369, 133–141. 10.1016/j.jphotochem.2018.10.027.

[ref125] Jangam K.; et al. Synthesis and characterization of magnetically separable Zn1-xCoxFeMnO4 nanoferrites as highly efficient photocatalyst for degradation of dye under solar light irradiation. J. Phys. Chem. Solids 2021, 148, 109700 10.1016/j.jpcs.2020.109700.

[ref126] Anandkumar M.; et al. Single-phase Gd0.2La0.2Ce0.2Hf0.2Zr0.2O2 and Gd0.2La0.2Y0.2Hf0.2Zr0.2O2 nanoparticles as efficient photocatalysts for the reduction of Cr(VI) and degradation of methylene blue dye. J. Alloys Compd. 2021, 850, 156716 10.1016/j.jallcom.2020.156716.

[ref127] Anandkumar M.; et al. Single-phase Gd0. 2La0. 2Ce0. 2Hf0. 2Zr0. 2O2 and Gd0. 2La0. 2Y0. 2Hf0. 2Zr0. 2O2 nanoparticles as efficient photocatalysts for the reduction of Cr (VI) and degradation of methylene blue dye. J. Alloys Compd. 2021, 850, 156716 10.1016/j.jallcom.2020.156716.

[ref128] Bhattacharya D.; et al. Visible light driven degradation of brilliant green dye using titanium based ternary metal oxide photocatalyst. Results in Physics 2019, 12, 1850–1858. 10.1016/j.rinp.2019.01.065.

[ref129] Li X.; et al. Novel Ag2ZnGeO4 photocatalyst for dye degradation under visible light irradiation. Appl. Catal. A: General 2008, 334 (1–2), 51–58. 10.1016/j.apcata.2007.09.033.

